# New-generation geostationary satellite reveals widespread midday depression in dryland photosynthesis during 2020 western U.S. heatwave

**DOI:** 10.1126/sciadv.adi0775

**Published:** 2023-08-02

**Authors:** Xing Li, Youngryel Ryu, Jingfeng Xiao, Benjamin Dechant, Jiangong Liu, Bolun Li, Sungchan Jeong, Pierre Gentine

**Affiliations:** ^1^ Research Institute of Agriculture and Life Sciences, Seoul National University, Seoul, South Korea.; ^2^ Department of Landscape Architecture and Rural Systems Engineering, College of Agriculture and Life Sciences, Seoul National University, South Korea.; ^3^ Earth Systems Research Center, Institute for the Study of Earth, Oceans, and Space, University of New Hampshire, Durham, NH, USA.; ^4^ German Centre for Integrative Biodiversity Research (iDiv) Halle-Jena-Leipzig, Leipzig, Germany.; ^5^ Leipzig University, Leipzig, Germany.; ^6^ Department of Earth and Environmental Engineering, Columbia University, New York, NY, USA.

## Abstract

Emerging new-generation geostationary satellites have broadened the scope for studying the diurnal cycle of ecosystem functions. We exploit observations from the Geostationary Operational Environmental Satellite-R series to examine the effect of a severe U.S. heatwave in 2020 on the diurnal variations of ecosystem photosynthesis. We find divergent responses of photosynthesis to the heatwave across vegetation types and aridity gradients, with drylands exhibiting widespread midday and afternoon depression in photosynthesis. The diurnal centroid and peak time of dryland gross primary production (GPP) substantially shift toward earlier morning times, reflecting notable water and heat stress. Our geostationary satellite-based method outperforms traditional radiation-based upscaling methods from polar-orbiting satellite snapshots in estimating daily GPP and GPP loss during heatwaves. These findings underscore the potential of geostationary satellites for diurnal photosynthesis monitoring and highlight the necessity to consider the increased diurnal asymmetry in GPP under stress when evaluating carbon-climate interactions.

## Teaser

Geostationary satellite reveals the asymmetrical impact of heatwaves on plant diurnal photosynthesis at the continental scale.

## INTRODUCTION

Over recent decades, our Earth has experienced a notable increase in record-breaking high temperatures (*1*, *2*), with the western United States emerging as a climatic “hot spot.” This region has endured recurring drought and heatwave events since the mid-2010s (*3*–*5*), resulting in dire consequences for both natural and human systems, including unprecedented water shortages, increased wildfires, substantial agricultural losses, and heightened human mortality (*6*, *7*). Dominated by water-limited dryland ecosystems (*8*), the western United States, particularly the Southwest, faces exacerbated water stress due to more frequent and protracted droughts and heatwaves. Such conditions can profoundly impair or even suppress ecosystem photosynthesis and carbon uptake, ultimately influencing the global carbon cycle’s interannual variability (*9*, *10*).

Investigating vegetation photosynthesis at various temporal scales offers valuable insights into vegetation growth, carbon uptake, and environmental interactions. While longer time scales (e.g., monthly, seasonal, and annual) reveal variations in photosynthesis influenced by vegetation phenology, weather/climate, and nutrient availability, photosynthesis at shorter scales (i.e., subdaily) is mainly affected by solar radiation and other environmental factors such as temperature, soil moisture, and vapor pressure deficit (VPD) that modulate plant function, particularly stomatal conductance (*11*–*13*). Over the past 30 years, ecosystem-level vegetation photosynthesis [i.e., gross primary production (GPP)] has been inferred from polar-orbiting satellite observations, such as Landsat, the Moderate Resolution Imaging Spectroradiometer (MODIS), and the Orbiting Carbon Observatory 2 (OCO-2) (*14*–*18*). However, these satellites, with their daily to multiday observation intervals, are adept at monitoring GPP at longer scales but limited in capturing diurnal variations (*19*, *20*). Consequently, direct interactions between photosynthesis and environmental factors at subdaily scales (e.g., “midday depression”) can be obscured or averaged out when aggregating instantaneous variables to daily or longer time scales (*21*). Fortunately, in recent years, emerging subdaily Earth observations have been available from several satellites and instruments (*21*), including new-generation geostationary satellites (*22*) and the ECOsystem Spaceborne Thermal Radiometer Experiment on Space Station (ECOSTRESS) (*13*, *23*) and the OCO-3 (*24*) on board the International Space Station. These innovative satellite observations present unparalleled opportunities to study diurnal variations in vegetation photosynthesis and their response to the environmental conditions over the course of a day at large spatial scales (*21*, *25*, *26*).

In contrast to ECOSTRESS and OCO-3 observations, which are spatially and temporally sparse and not continuous throughout the day (*13*, *27*, *28*), new-generation geostationary satellites such as the Geostationary Operational Environmental Satellite-R (GOES-R) and Geostationary Korea Multi-Purpose Satellite-2A offer high-frequency observations (ranging from several minutes to hourly) of radiance, surface reflectance, and land surface temperature (LST) at moderate spatial resolutions (1 to 3 km). This has facilitated groundbreaking research that transcends traditional applications of polar-orbiting satellites, including enhanced monitoring of vegetation seasonality in the cloud-covered Amazon (*29*), investigation of diurnal behavior of urban heat island (*30*) and wildfires (*31*), and mapping of photosynthesis at various times of day (*21*, *26*). However, no studies have yet harnessed geostationary satellite observations to monitor diurnal variations in vegetation photosynthesis in relation to droughts or heatwaves on a broad spatial scale.

Here, we estimate hourly GPP across the conterminous United States (CONUS) based on GOES-R observations along with other ancillary inputs and then investigate how the diurnal cycle of photosynthesis responds to the severe late-summer heatwave of 2020 (fig. S1). This heatwave affected nearly the entire western United States, encompassing both water-sensitive dryland ecosystems (fig. S2) and more drought-resilient ecosystems, offering a valuable opportunity to examine their potentially divergent responses. This study offers a comprehensive exploration of heatwave impacts on the diurnal dynamics of photosynthesis at a continental scale. Our findings reveal a widespread midday and afternoon depression of photosynthesis in dryland ecosystems during the heatwave, a phenomenon not discernible through polar-orbiting satellite observations. We investigate the environmental regulation of diurnal photosynthesis dynamics across diverse ecosystems and elucidate how current methods for upscaling polar-orbiting satellite snapshots to daily means may under- or overestimate daily GPP.

## RESULTS

### Widespread midday and afternoon depression in ecosystem photosynthesis during the heatwave

We first estimate hourly GPP across the CONUS using a machine learning method driven by GOES-R observations and other gridded variables including LST, shortwave incoming radiation (SW), VPD, normalized difference vegetation index (NDVI), and land cover type. From this, we derive three diurnal metrics: the diurnal centroid of GPP (*C*_GPP_), GPP peak hour (Hour_peak_), and the ratio of afternoon GPP to morning GPP (Ratio_A/M_; Materials and Methods). We then calculate the difference between the heatwave year 2020 and two preceding more regular years (2018 and 2019) and refer to this difference as “anomaly.”

**Fig. 1.**
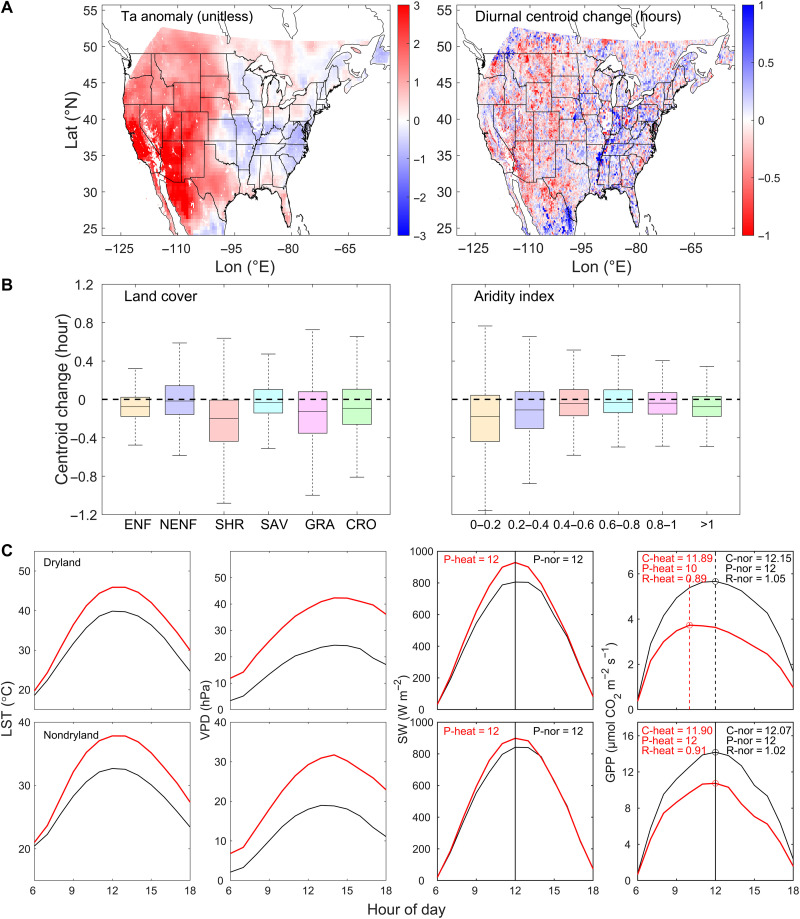
Changes in environmental variables, gross primary production, and diurnal metrics during the heatwave from 14 to 19 August 2020 relative to 2018 and 2019 across the CONUS. (**A**) Standardized normalized anomalies of air temperature (Ta_ano, unitless) from MERRA-2 and diurnal centroid changes (units: hours) from 14 to 19 August 2020 relative to the multiyear average. (**B**) Different responses of diurnal metrics to the heatwave across vegetation types and along aridity gradients [smaller aridity index (AI) values indicate more arid conditions]. ENF, NENF (or non-ENF), SHR, SAV, GRA, and CRO represent evergreen needleleaf forest, other forests except for ENF, shrubland, savanna, grassland, and cropland, respectively. Box plots illustrate the distribution of diurnal change: The box represents the interquartile range (IQR), containing data from the 25th percentile (Q1) to the 75th percentile (Q3). The horizontal line inside the box indicates the median (50th percentile). The whiskers extend to the minimum and maximum values within 1.5 times the IQR from Q1 and Q3, respectively. The outliers beyond this range are plotted as individual red plus symbols. (**C**) Regional-mean hourly land surface temperature (LST), vapor pressure deficit (VPD), shortwave incoming radiation (SW), and gross primary production (GPP) for drylands and nondrylands in normal (black) and heatwave (red) years. C-heat, P-heat, and R-heat represent the diurnal centroid of GPP (*C*_GPP_), GPP peak hour (Hour_peak_), and the ratio of afternoon GPP to morning GPP (Ratio_A/M_) during the heatwave, while C-nor, P-nor, and R-nor represent these metrics in the normal years. The hours mentioned here correspond to local time.

The 2020 anomaly maps of the three diurnal metrics reveal a widespread midday and afternoon depression in ecosystem photosynthesis during the heatwave in the western United States (Fig. 1A and fig. S3). *C*_GPP_ and Hour_peak_ shift toward earlier morning for the majority of the western regions, and the Ratio_A/M_ also shows a marked decline. For example, for dryland regions experiencing a standardized normalized air temperature anomaly (henceforth “Ta_ano”) larger than 1 (fig. S2), 66.9% exhibit morning-shifted *C*_GPP_. In some dryland regions where the diurnal cycle of GPP was asymmetrical in the normal years (fig. S4), the heatwave further suppresses photosynthesis from noon onward and leads to increased diurnal asymmetry in GPP. The shift in diurnal metrics positively correlates to the daily GPP change, implying that the morning shift in diurnal metrics generally results in a decrease in daily total GPP (fig. S5).

The diurnal metrics exhibit divergent responses to the heatwave across vegetation types and along aridity gradients (Fig. 1B and fig. S3). Overall, shrubland and grassland are more sensitive to the heatwave than the other vegetation types (e.g., forest, savanna, and cropland). Among forests, only the evergreen needleleaf forest (ENF) sees a systematic shift in *C*_GPP_ and Hour_peak_, while the other forests are more resistant to the heatwave, maintaining relatively stable diurnal cycles. The impact of the heatwave on the diurnal cycle of photosynthesis is predominantly observed in arid and semiarid regions with an aridity index (AI) below 0.6, and the shift becomes weaker as the AI increases (toward more humid conditions). The findings related to the widespread midday and afternoon reduction in ecosystem photosynthesis, along with the differing diurnal metric responses to heatwaves across various vegetation types and aridity gradients, remain almost unchanged when the baseline period for calculating the mean of variables under normal conditions is expanded to 2018–2022 (Materials and Methods; fig. S6). Similarly, these findings stay consistent when different methods are used to calculate the centroid shift (fig. S7).

The regional-mean diurnal course of environmental variables and GPP (Fig. 1C) shows that, as expected, both LST and VPD are notably elevated during the heatwave compared to normal years. For drylands, the intensified heat condition leads to a GPP peak time occurring 2 hours earlier and, correspondingly, morning-shifted *C*_GPP_ and Ratio_A/M_. In contrast, the diurnal metrics of nondryland regions exhibit only minor changes. The higher morning-time GPP of drylands and the resistance of nondryland ecosystems to the heat condition are further confirmed by eddy covariance (EC) data from flux towers (figs. S8 and S9) and using the original GOES LST without gap-filling (fig. S10). Figure S11 provides a representative example of the diurnal course of environmental and vegetation variables at four sites with different vegetation types, based on EC observations. More dryland sites with earlier occurrence of GPP peak hour during heatwaves are provided in fig. S12. These site-level observations are consistent with our regional-level findings derived from gridded GPP estimates.

In the event that the impact of a heatwave on ecosystem photosynthesis was consistent and uniform across hours throughout the day, the largest loss of GPP during the heatwave would be expected at the time when vegetation had maximum productivity. However, our findings reveal a substantial down-regulation of photosynthesis from noon onward, leading to the largest GPP loss at noon or during the afternoon for the majority of western regions. Notably, this timing occurs later than the GPP peak hour during the heatwave year for 72.6% of dryland regions (fig. S13), further substantiating the asymmetric influence of heatwaves on diurnal photosynthesis fluctuations.

### Environmental controls on diurnal behavior of ecosystem photosynthesis

**Fig. 2.**
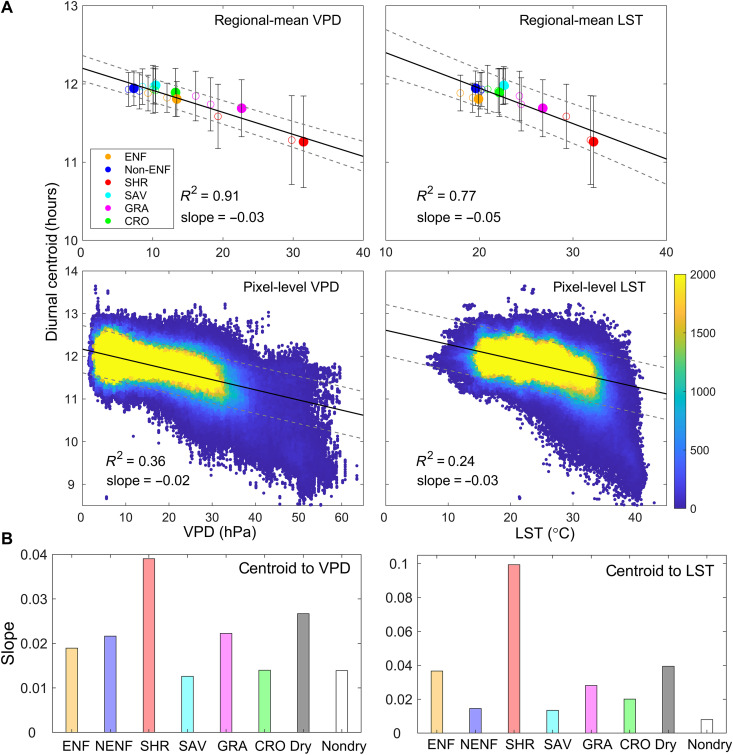
Relationship between diurnal centroid (*C*_GPP_) and daily VPD (or LST). (**A**) Regional-mean (first row) and pixel-level (second row) relationships between *C*_GPP_ and VPD (or LST). Filled circles represent the heatwave year, and hollow circles represent normal years with error bars indicating the SD of *C*_GPP_. The solid line represents the best-fit line derived from linear regression analysis, and two dashed lines represent the 95% confidence interval for the regression estimate. There are 18 circles in the first row (3 years multiplied by six vegetation types). (**B**) Illustrates the slope of VPD (or LST)–*C*_GPP_ linear relationship for different vegetation types and for drylands or nondrylands. NENF (or non-ENF) represents other forests, excluding ENF. The units of slope are hour/hPa and hour/°C, respectively.

We examine the controls of different environmental factors on diurnal variations of ecosystem photosynthesis at both regional and pixel levels (Materials and Methods). The regional-mean daily VPD and LST show strong negative relationships with regional-mean *C*_GPP_ (*R*^2^ = 0.91 and 0.77, *P* < 0.0001), suggesting that the increase of heat and water stress contributes to an earlier coming of *C*_GPP_ (Fig. 2A). At the pixel level, the negative relationships are still observed despite the weaker correlations (*R*^2^ = 0.36 and 0.24, *P* < 0.0001). The pixel-level relationships for different vegetation types (Fig. 2C and fig. S14) show that shrubland has the strongest negative relationships and largest negative slopes between *C*_GPP_ and VPD (*R*^2^ = 0.55, slope = −0.04 hour/hPa) or LST (*R*^2^ = 0.44, slope = −0.1 hour/°C) among all the vegetation types, followed by ENF and grassland. A 1-hPa increase in VPD leads to a 0.04-hour morning shift in shrubland *C*_GPP_, and a 1°C increase in LST advances *C*_GPP_ by 0.1 hour. Overall, the *C*_GPP_ of drylands exhibits twice the sensitivity (slope) to VPD variations and five times the sensitivity to LST variations compared to nondryland regions (VPD: −0.027 hour/hPa versus −0.014 hour/hPa; LST: −0.039 hour/°C versus −0.008 hour/°C; Fig. 2C and fig. S15). The relationships between regional-mean Hour_peak_ (or Ratio_A/M_) and daily VPD (or LST; fig. S16) are similar to those observed between *C*_GPP_ and VPD (or LST). Figure S17 further substantiates the consistency of the responses of the *C*_GPP_ to VPD and LST across the two baseline periods. Notably, the slope of the *C*_GPP_ to changes in VPD and LST is almost identical between the two baseline periods. The environmental controls on diurnal photosynthesis dynamics are further confirmed by EC flux tower observations (fig. S18), which indicates that diurnal metrics are more strongly regulated by VPD (*R*^2^ = 0.47 to 0.60, *P* < 0.001) and LST (*R*^2^ = 0.46 to 0.58, *P* < 0.001) than by Ta (*R*^2^ = 0.33 to 0.35, *P* < 0.001).

**Fig. 3.**
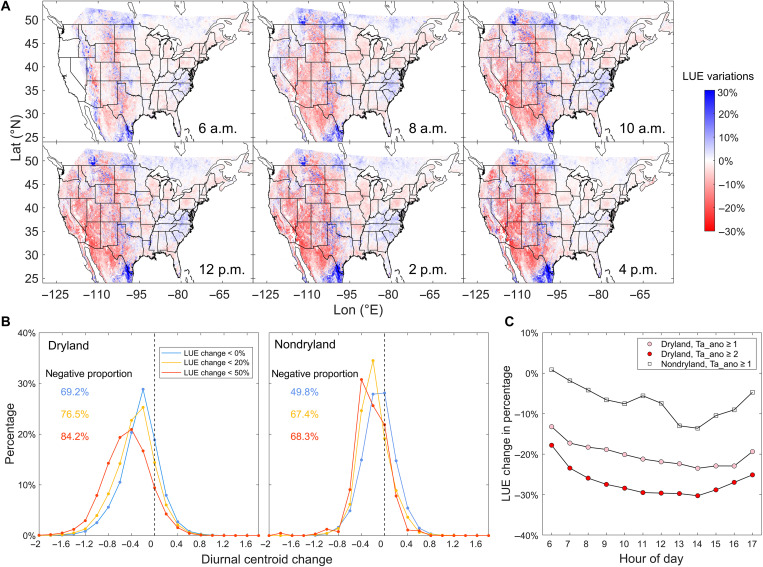
LUE variations during the heatwave. (**A**) Hourly change in LUE (expressed as a percentage) during the heatwave compared to normal years. (**B**) Probability distribution of diurnal centroid change in response to daily LUE decrease for three different situations: daily decrease of LUE ≤ 0, 20, and 50%, respectively. (**C**) Hourly LUE change over the course of the day for three different situations: drylands with Ta_ano ≥ 1 (pink circles); drylands with Ta_ano ≥ 2 (red circles), and nondrylands with Ta_ano ≥ 1 (black squares).

Because light use efficiency (LUE) reflects the impact of changes in the environment on plant photosynthesis without the strongly dominant solar radiation signal present in GPP, we calculate hourly LUE to examine how it varies over the course of a day during the heatwave (Materials and Methods; fig. S19). The spatial patterns of hourly LUE anomaly coincide with those of the GPP diurnal metrics (Fig. 3A), with widespread negative anomalies in the western United States. This indicates that the LUE changes contribute to the observed shifts in diurnal GPP metrics during the heatwave. As LUE decreases, 69.2% of drylands exhibit a morning shift in *C*_GPP_, with this proportion increasing to 76.5 and 84.2% for regions experiencing larger LUE reductions (<20 and <50%). In contrast, LUE decreases lead to less pronounced changes in *C*_GPP_ for nondryland regions (Fig. 3B). Figure 3C illustrates that, in nondrylands, the LUE only decreases by 0 to 12% over the course of a day, while in drylands, the hourly reduction in LUE is around 20% and intensifies as the heat stress increases (Ta_ano from 1 to 2). For both ecosystems, the most substantial LUE decline occurs in the early afternoon.

### Geostationary satellite-based method better estimates daily GPP and GPP loss during the heatwave

**Fig. 4.**
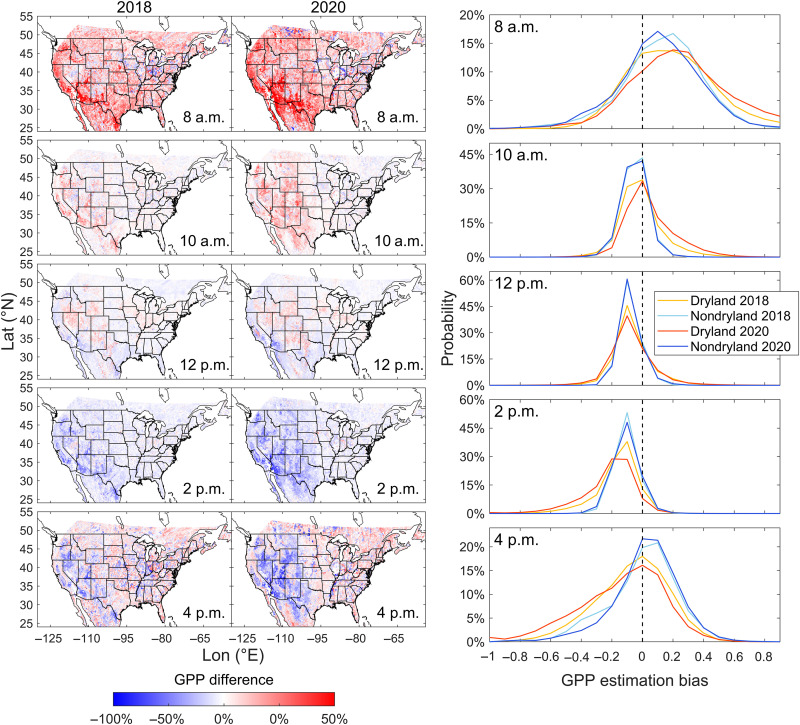
Difference of daily GPP based on upscaling from single hours: 8 a.m. to 4 p.m. and aggregated from hourly GPP based on GOES-R during 14 to 19 August in both normal and heatwave years. Difference is calculated as (GPP_upscaling_ − GPP_GOES_)/GPP_GOES_. The red/blue pixels indicate over/underestimation of daily GPP based on the upscaling method compared to GPP_GOES_. The third column shows the probability distribution of GPP over/underestimations for drylands and nondrylands in both years.

We first compare the daily GPP upscaled from a single hour (GPP_upscaling_) based solely on radiation, which emulates polar-orbiting satellites, with daily GPP aggregated from our hourly GPP derived from GOES-R (GPP_GOES_; Materials and Methods). Figure 4 shows the spatial difference maps between daily GPP_upscaling_ upscaling from GPP at either 8 a.m., 10 a.m., 12 p.m., 2 p.m., or 4 p.m. and daily GPP_GOES_ for both normal and heatwave years. The results clearly demonstrate that, for both years, using a snapshot from the earlier part of the morning (e.g., 8 a.m.) and solely considering radiation variations for daily upscaling (*19*) lead to an overestimation of daily GPP across the majority of the United States. Conversely, using an afternoon observation for daily upscaling (e.g., 2 p.m.) results in an underestimation of daily GPP. The upscaling method relies only on radiation and does not account for changes induced by varying environmental stresses and LUE throughout the day. Similar results are observed when using fixed hourly LUE to estimate daily GPP (fig. S20). Notably, these errors are also widespread in the normal year but become even more pronounced during the heatwave year.

**Fig. 5.**
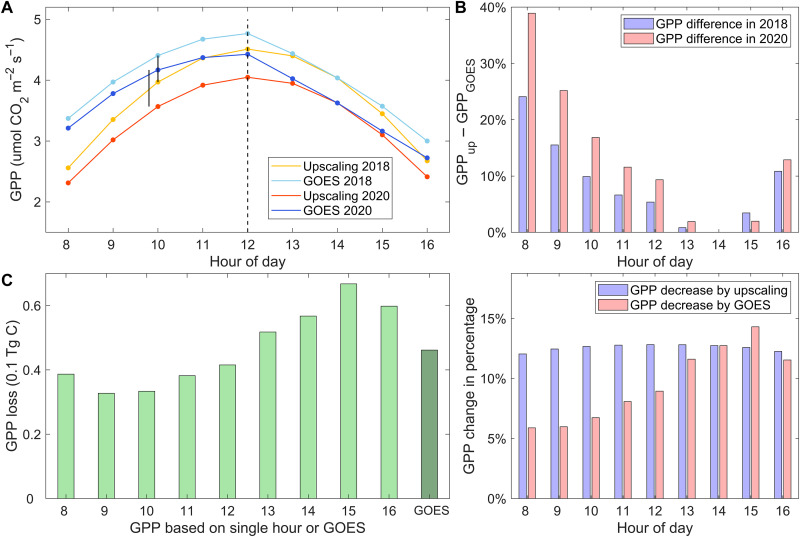
Difference in estimating regional-mean hourly GPP and daily GPP loss using the upscaling-based method (GPP_upscaling_) and the GOES-based method (GPP_GOES_). (**A**) Regional-mean hourly GPP based on the two methods during 14 to 19 August in both normal and heatwave years under Ta_ano ≥ 1. The difference between two GPPs is larger in 2020 (longer vertical black line) than that in 2018 (shorter vertical black line). (**B**) Difference between GPP_upscaling_ and GPP_GOES_ calculated as (GPP_upscaling_ − GPP_GOES_)/GPP_GOES_ in both years for each hour (upper) and the resulting biases propagated to the calculation of GPP loss during the heatwave (lower) under Ta_ano ≥ 1. For example, at 8 a.m., GPP_upscaling_ underestimates GPP_GOES_ by 24% in the normal year but by 39% in the heatwave year. When calculating GPP loss, GPP_GOES_ only detects about a 5% decrease in GPP at 8 a.m., while GPP_upscaling_ overestimates the GPP loss (~12% decrease). (**C**) Regional total daily GPP loss from 14 to 19 August during the heatwave year based on two methods. Light green bars denote GPP loss based on GPP_upscaling_ from different hours: 8 a.m. to 4 p.m., respectively, and the dark green bar denotes GPP loss based on GPP_GOES_. The hours mentioned here correspond to local time.

Biases stemming from the radiation-based upscaling method can also affect the calculation of GPP difference between normal and heatwave years (Fig. 5). The upscaling-based regional-averaged GPP (here, using 2 p.m. as an example) exhibits highly symmetric diurnal cycles and underestimates GPP_GOES_ with asymmetrical diurnal cycle in both years, primarily during morning hours (Fig. 5A). The underestimation is more pronounced in the heatwave year, resulting in an overestimation of heat-induced GPP loss during morning hours and, consequently, an overestimation of daily GPP loss (Fig. 5B). As heat conditions and diurnal asymmetry in GPP intensify, the underestimation of GPP_upscaling_ relative to GPP_GOES_ and the overestimation of heat-induced GPP loss using GPP_upscaling_ both largely increase (fig. S21). We further calculate the regional total daily GPP for drylands from 14 to 19 August for normal and heatwave years. Both daily GPP_upscaling_ from all single hours and GPP_GOES_ capture the decline of dryland productivity during the heatwave (Fig. 5D). The estimated GPP loss for the entire region during the heatwave period based on GOES-R is approximately 0.4 Tg C per day, while GPP loss based on upscaling from different hours ranges from 0.25 to 0.6 Tg C per day.

## DISCUSSION

With the recent launch of new-generation satellites capable of diurnal sampling, several pioneering studies have explored their potential for monitoring the diurnal cycle of photosynthesis (*13*, *21*, *26*, *32*, *33*). Our hourly GPP estimates, based on GOES-R satellite observations, effectively capture the diurnal dynamics of ecosystem photosynthesis in response to heatwaves at the regional scale. We present observational evidence of an earlier occurrence of dryland GPP peak hour during the heatwave due to widespread midday and afternoon depression in photosynthesis. Numerous studies based on polar-orbiting satellites have extensively investigated the effects of droughts and heatwaves on seasonal and interannual variations of photosynthesis (*34*–*36*). However, diagnosing the interactions between photosynthesis and environmental factors at the subdaily scale was previously limited to site-level investigations based on flux towers or proximal remote sensing observations (*11*, *37*–*39*). One earlier study attempted to generate global monthly averaged half-hourly GPP by upscaling site-level half-hourly GPP data using machine learning methods (*40*), but this approach could not track the diurnal dynamics of photosynthesis for each day. A recent study (*21*) used OCO-3 solar-induced chlorophyll fluorescence (SIF) data based on the snapshot area mode (SAM) for the first time to demonstrate that a heatwave in Australia led to a decline in plant photosynthesis in the afternoon. OCO-3 SAM data, however, are only available in a very limited number of areas across the globe and thereby do not allow for studies over broad spatial domains like the CONUS. Another recent study also revealed the afternoon depression of dryland photosynthesis based on SIF from OCO-3 during the drought, but the real change in diurnal photosynthesis dynamics could not be quantified because of the sparse and discontinuous OCO-3 measurements (*41*). With the high frequency of GOES-R observations, we generate hourly GPP maps for all days during the heatwave across the CONUS at a high spatial resolution (~0.025°). We reveal an asymmetrical diurnal response of GPP to environmental stresses across a wide range of dryland ecosystems in the western United States, and the peak time of GPP shifts further toward earlier morning times during the severe heatwave. In contrast, OCO-3 SIF data particularly in nadir/glint mode are extremely sparse both spatially and temporally, and only relying on averaged morning or afternoon observations over large space and long time windows (e.g., 1° and monthly) ignores the spatial, hour-to-hour and day-to-day variations within those windows (*41*). GOES-R observations further enable us to quantify how much the increase of VPD and LST led to a morning shift in GPP diurnal metrics and, subsequently, heatwave-induced loss of GPP, which is not possible using current OCO-3 SIF data.

The midday or afternoon depression in dryland photosynthesis has been reported at the site level using GOES-R or ECOSTRESS data (*13*, *32*) and at the regional scale using satellite SIF (*21*, *33*, *41*, *42*). This depression has a crucial effect on integrated daily photosynthesis and, ultimately, the accumulated vegetative biomass of drylands (*43*). Although drylands are considered better adapted to high temperatures and water-deficit conditions, heatwave events with increasing persistence and severity are pushing these ecosystems beyond their historical regimes. The frequent and widespread earlier depression of carbon uptake could result in a higher risk of hydraulic failure (*12*). The way in which the ongoing global warming alters the climatic responses of dryland photosynthesis across time scales requires further investigation (*44*, *45*). To that end, emerging efforts have been made recently by using advanced techniques, observational platforms, and datasets to better understand the water-carbon coupling in drylands from subdaily to interannual scales (*21*, *33*, *40*, *41*, *46*, *47*). Our study further reveals the increased diurnal asymmetry of dryland photosynthesis during heatwaves and the contrasting diurnal responses of nondryland ecosystems to heat stress. By quantifying the ecosystem-specific sensitivity of diurnal photosynthesis to environmental factors, such as LST and VPD, our study can contribute to the benchmarking of land surface models at the subdaily scale. In addition, it may help predict how the diurnal cycle of GPP will respond to climate change in the near future.

The widespread depression of dryland GPP from noon onward highlights the importance of considering the asymmetrical diurnal cycle when upscaling photosynthesis from the hourly to daily scale. Neglecting this asymmetry can lead to substantial biases in daily total GPP estimates (*40*). This underscores the value of geostationary satellites, while also raising concerns about studies that examine the response of dryland photosynthesis to climate based on snapshots from polar-orbiting satellites. Currently, there are generally two ways for generating daily estimation of photosynthesis (such as GPP and SIF): using daily-averaged environmental drivers (*14*, *15*) or upscaling instantaneous photosynthesis observations from polar-orbiting satellites by assuming an ideal symmetrical radiation pattern (with a peak at solar noon) over the course of the day (*17*, *19*, *48*, *49*). Both approaches may introduce additional biases. For GPP models driven by daily-mean meteorology, the averaging effect can lead to a misinterpretation of environmental regulation on photosynthesis. For instance, if high temperatures only reduce midday GPP rather than daily mean GPP, using daily-mean GPP and temperature may not reveal the true interaction between climate and ecosystems. For daily photosynthesis estimates based on upscaling methods, biases can be largely offset by using both morning and afternoon observations as did in BESS model (*16*), which used the average of GPP derived from both Terra (10:30 a.m.) and Aqua (1:30 p.m.), but are inevitable for current satellite SIF data because the daily SIF is converted from snapshots either in the morning (GOME-2) or at midday (GOSAT, OCO-2, and TROPOMI) (*17*, *48*). Therefore, when using satellite SIF data to explore the environmental responses of photosynthesis, weakened or enhanced responses should not be directly interpreted as the internal response of ecosystems.

Our study demonstrates the potential of incorporating subdaily environmental information from geostationary satellites to monitor diurnal photosynthesis dynamics under stress. Our methods can be extended to different regions globally as more and more observations from new-generation geostationary satellites or global continuous products become available, such as GeoNEX, a collaborative effort from global geostationary satellite sensors (*26*, *50*, *51*). Furthermore, our approach for estimating hourly GPP can also be applied to estimating hourly evapotranspiration (ET) and water use efficiency (*21*, *52*). This would help gain insight into changes in how plants use water at the subdiurnal time scales and its response to environmental factors. Addressing these critical scientific questions will become increasingly valuable as global warming intensifies. In the near future, the possibly distinct influences of heatwaves on the diurnal dynamics of photosynthesis at different phenological stages of vegetation (e.g., green-up and senescence) can be further explored. In addition, as heatwaves will continue to occur, it is essential to evaluate whether the morning shift of the diurnal cycle of dryland photosynthesis will become more pronounced and whether an irreversible shift poses a risk of vegetation mortality. Upcoming missions [e.g., TEMPO (*53*) and Sentinel-4 (*54*)] that may provide temporally frequent and continuous observations of photosynthesis proxies (e.g., SIF) hold potential to further enrich our understanding and research of dryland photosynthesis at the subdaily scale.

## MATERIALS AND METHODS

### Severe U.S. heatwave in August 2020

The western part of the United States, especially the Southwest, has frequently been hit by heatwaves and droughts in recent years (*3*). In mid-August 2020, a severe heatwave occurred over the majority of the U.S. states, with standardized normalized anomaly of daily temperature approaching a value of 5 in California (figs. S1 and S2). This heatwave coincided with a record-high precipitation deficit, and it mainly affected the western United States, with southwestern states including Arizona, Nevada, Utah, Colorado, and New Mexico experiencing the hottest conditions. According to one recent study, this exceptional heat and atmospheric dryness led to a large loss of local ecosystem productivity (*55*). Here, we selected 14 to 19 August 2020, as the heatwave period (figs. S1 and S2) to examine how the heatwave affected the diurnal dynamics of ecosystem photosynthesis. Notably, the hot conditions started as early as June for many regions in the Southwest. The heatwave in the selected period spread to more widespread regions including California with extreme heat conditions.

### Model training and evaluation

We used a Cubist regression tree model (*56*) to predict half-hourly or hourly GPP. The Cubist model creates a series of rules between the target variable *GPP* and the explanatory variables, where their relations are explicitly expressed in multivariate linear models. Cubist helps find the corresponding rules that explanatory variables match and generates predictions based on these rules. Sometimes, Cubist creates multiple predictions when more than one rule matches, and the average value of these predictions is used to determine the final prediction. The Cubist model has been successfully used in previous studies to predict carbon-related variables including net ecosystem carbon exchange (NEE), GPP, and SIF (*13*, *18*, *57*). More details on the Cubist model can be found in these studies.

Three types of explanatory variables were considered for predicting GPP in this study, including three environmental variables (LST, SW, and VPD), one vegetation variable [vegetation indices such as NDVI, the enhanced vegetation index (EVI) or the near-infrared reflectance of vegetation (NIRv)], and one categorical variable (land cover type; table S1). The five selected variables were considered to have close relationships with GPP, and the roles of environmental variables in regulating the diurnal variations of EC GPP has been demonstrated in one of our previous studies (*13*).

Half-hourly or hourly VPD and SW were obtained from EC flux towers from 77 AmeriFlux sites (table S2). The sites were selected on the basis of their data availability covering August 2020 and their homogeneity, which was determined by the consistency between the land cover type of the site and the dominant land cover of the 0.05° grid cell. The land cover type was determined on the basis of the site description. The REddyProc software (*58*) was used to fill the gaps in the EC data. In this study, to partition NEE into GPP, we used both daytime (*59*) and nighttime partitioning methods (*60*). The samples with differences of the GPP estimates based on the two methods over 10 μmol CO_2_ m^−2^ s^−1^ were excluded from the training. The LST was obtained from GOES-16 (*61*), which is the first mission of the GOES-R series and was launched by the National Oceanic and Atmospheric Administration in November 2016. The hourly GOES-16 LST was then extracted for each site at the grid cell where the site was located.

We extracted and calculated three vegetation indices (NDVI, EVI, and NIRv) for each site from the daily MODIS bidirectional reflectance distribution function (BRDF)–corrected reflectance product MCD43A4 (Collection 6, 500 m) through MODIS and VIIRS Land Products Global Subsetting and Visualization Tool (ORNL, 2018). This was performed differently from a recent study (*32*) that converted the top-of-atmosphere radiances from GOES-16 to top-of-atmosphere reflectance and derived the BRDF-corrected surface reflectance using the radiative transfer model and BRDF model for two reasons. First, the heatwave in August 2020 mainly hit the dryland ecosystems. During the heatwave, the diurnal variations of GPP are mainly controlled by solar radiation and environmental factors (such as VPD and air temperature). The contribution of the canopy structure to the diurnal GPP during short time scales is assumed to be much smaller than that of physiological changes (*62*). Second, MODIS provides operational BRDF-corrected reflectance products that have been validated across temporal and spatial scales and different ecosystems (*63*), while GOES-R requires further efforts to produce and comprehensively validate its BRDF products, which is beyond the scope of the current study.

In total, we obtained more than 500,000 half-hourly or hourly samples from May 2017 to December 2020 for 77 AmeriFlux sites (table S2). Only flux data (GPP, VPD, and SW) with a good quality flag equal to 0 or 1 (0, original; 1, most reliable) were used for model development, and for MODIS vegetation indices, only the observations with a quality flag equal to 0, indicating good quality and full BRDF inversion, were used. For training, we first sorted the samples based on the LST values and then divided them into 14 bins ranging from −10° to 60°C with 5°C interval. In each bin, we randomly selected two-thirds of the data as training samples, and the remaining one-third as testing data. Compared with the completely random selection method, this processing ensured that both the low and high values of LST could be uniformly sampled and that the LST distribution in the training and testing datasets was consistent. We also tested the performance of GPP predictions in each LST bin by increasing the proportion of samples with higher LST (e.g., ≥30°C). In addition, we further assessed the model performance using the leave-one-out validation method to provide a more objective evaluation of our model. For each land cover type, we randomly selected data from one site as test data and used data from the remaining sites as training samples. We randomly repeated the training and validation process 200 times and calculated the mean and SD of the model’s performance metrics [*R*^2^ and root mean square error (RMSE)].

Table S4 shows the statistical measures for model evaluation with GPP derived from daytime-based method (GPP_day_). We found that the model including vegetation index, land cover, and three environmental variables (LST, SW, and VPD) performed the best in estimating the hourly GPP (*R*^2^ = 0.88, RMSE = 2.51 μmol CO_2_ m^−2^ s^−1^). When the data from the same sites were not used for training, the model still performed well, with an *R*^2^ of 0.82 ± 0.06 and an RMSE = 2.59 ± 0.63 μmol CO_2_ m^−2^ s^−1^ (fig. S22). Among the five variables, vegetation index and SW were two of the most important variables for GPP predictions, and excluding either of them substantially reduced the accuracy of model prediction. Including either/both of GOES LST or/and VPD could only slightly improve the GPP predictions (for all the samples) compared to the model solely based on SW. However, the model with either/both the VPD or/and LST included performed much better for the samples with higher LST (fig. S23). For example, for samples with LST ≥ 40°C, compared with the model including both VPD and LST, the models without either/both showed a decrease in *R*^2^ by 0.05 to 0.18 and increase in relative RMSE (rRMSE) by 0.08 to 0.15. The model solely based on radiation was unable to account for the effects of water and heat stress on photosynthesis. The three vegetation indices (NDVI, EVI, and NIRv) showed comparable performance for estimating GPP. In the following analyses, we only provide the results from NDVI because it is a simple and most widely used vegetation index, and previous studies also showed its smaller diurnal variations and lower sensitivity to BRDF effects compared to EVI or NIRv (*64*, *65*).

The model performance based on GPP derived from a nighttime-based method (GPP_night_) is provided in table S5. The findings regarding the variable importance were similar to those based on GPP_day_ but with lower prediction performance. For example, the RMSE of the same model consisting of VPD, SW, NDVI, LST, and land cover increased by 12% based on GPP_night_. Therefore, we used the model based on GPP_day_ to predict regional-level hourly GPP. The results also showed that changing the proportion of samples with higher LST had little effect on the GPP predictions (table S5), and only negligibly improved the prediction for samples with higher LST (not shown). The scatterplots of predicted GPP against EC GPP (GPP_day_) are shown in fig. S24 separately for different vegetation types.

### Regional mapping of hourly GPP

Once the predictive hourly GPP model was established at the site level, we then applied it to the regional scale to estimate hourly GPP across the CONUS driven by explanatory variables from gridded products (table S3). We generated hourly GPP maps in August from 2018 to 2020 (missing data of SW across our study region in 2017) at a spatial resolution of 0.025°.

The hourly SW (ABI-L2-DSRC product, 0.25°) and LST (ABI-L2-LSTC product, ~ 2 km) were obtained from GOES-16. Information on the algorithms for generating these data from geostationary satellites, as well as the validation and uncertainty analysis, can be found in related studies (*25*, *61*). The vegetation indices were derived and calculated from 0.05°, daily MODIS BRDF-corrected reflectance products (MCD43C4). The land cover map in 2020 was obtained from 0.05° MODIS land cover products (MCD12C1) with the International Geosphere-Biosphere Programme (IGBP) classification scheme. Because of the short study period (2018–2020), we did not consider the land cover change. The hourly VPD was obtained from the ERA5-land reanalysis dataset (0.1°) (*66*). We compared the GOES SW and ERA5 VPD against EC observations for each site (fig. S25) and found that the GOES SW and ERA5 VPD were highly correlated with EC SW and VPD (median *R*^2^ = 0.83, RMSE = 115 W/m^2^ for SW; median *R*^2^ = 0.80, RMSE = 4.7 hPa for VPD). The errors between gridded products and site observations were slightly larger than those in our previous study (*13*) because we only used samples in August in this study, including more samples throughout the year with strong seasonal variations could improve the *R*^2^. The coarser resolution of GOES SW and ERA5 VPD relative to the footprint of EC flux towers could also have increased their inconsistency. We resampled SW, VPD, and vegetation indices to 0.025° by bilinear interpolation and land cover map to 0.025° by nearest-neighbor interpolation to match the spatial resolution of GOES-16 LST.

Although GOES-16 provided many more LST observations per day compared to polar-orbiting satellites such as MODIS, the existing cloud cover still affected the data availability of LST (*27*). Therefore, we used LST from ERA5-land products (0.1°) (*66*) to fill the gaps in GOES-16 LST data. We extracted both LST observations for the 77 sites and established the linear relationships between the two LST observations per hour for each land cover type (fig. S26). When there were valid GOES-16 LST observations, we used them for regional GPP predictions, while when they were missing because of clouds, we then used filled LST on the basis of ERA5 for GPP predictions. Last, we generated spatially and temporally continuous hourly GPP. To examine the diurnal variations of GPP during the heatwave, we relied on the gap-filled GPP and also compared the results generated from the original GOES LST without gap-filling. We hypothesize that we can also reveal the impact of the heatwave on diurnal GPP using the original GOES LST data, but its use will be limited because of the spatial discontinuity. Auxiliary data from other sources (e.g., ERA5) can help overcome its limitation and enable more flexible applications.

Figure S27 shows an example of predicted hourly GPP from 6 a.m. to 5 p.m. on 1 August 2018 (Pacific Daylight Time) across the CONUS. The ecosystems start photosynthesizing at about 6 to 7 a.m. (local time) when sunlight is available; GPP increases from early morning to noon with the increase of radiation and favorable environmental conditions (such as sufficient moisture) and then keeps decreasing in the afternoon until sunset. It also reveals contrasting productivity between the western part of the United States dominated by drylands and mesic ecosystems in the eastern part throughout the day. In the western United States, except for some forests along the coast, most arid and semiarid ecosystems that are less productive exhibited lower GPP over the course of a day, while in the eastern part, some ecosystems such as forests in the Appalachian Mountains maintained high photosynthetic activity from early morning to late afternoon. The most productive crops in the Corn Belt of the central United States were also captured by the hourly GPP maps.

### Impact of heatwave on diurnal cycles of GPP

First, we averaged hourly GPP during the 6-day heatwave period (14 to 19 August) for each year from 2018 to 2020. On the basis of the hourly GPP, we then derived three diurnal metrics for each pixel: the diurnal centroid, GPP peak hour, and the ratio of afternoon GPP to morning GPP. The diurnal centroid is often used to quantify the diurnal shifts in EC flux variables (NEE, GPP, or ET) induced by environmental conditions (*67*, *68*). For a given pixel, the diurnal centroid of GPP (*C*_GPP_) was defined as follows

$$C_{\mathrm{GPP}}=\frac{\sum ({\mathrm{GPP}}_{t}\times t)}{\sum {\mathrm{GPP}}_{t}}$$

where *t* is the time in decimal hours from 7 to 17 (7 a.m. to 5 p.m.) and GPP*_t_* is the GPP at hour *t*. The resulting *C*_GPP_ is the weighted mean hour of the diurnal cycle of GPP. For example, if *C*_GPP_ is greater than 12, then it indicates a shift of GPP toward afternoon, while if *C*_GPP_ is less than 12, then it indicates a shift toward morning (*32*). The GPP peak hour (Hour_peak_) was defined as the time at which GPP reached the maximum from 7 a.m. to 5 p.m. The ratio of afternoon GPP to morning GPP (Ratio_A/M_) indicated the difference between the averaged GPP in the afternoon (1 p.m. to 5 p.m.) relative to that in the morning (7 a.m. to 11 a.m.). For pixels, if the GPP at 7 a.m. or 5 p.m. was missing, then the GPP in the afternoon and morning was then averaged from shorter times (1 p.m. to 4 p.m. and 8 a.m. to 11 a.m., respectively). These three diurnal metrics adequately describe the diurnal pattern (symmetry or asymmetry) of GPP during the heatwave. Because we aimed to examine the effect of water and heat stress on diurnal GPP, we had to isolate any shift or variations of these diurnal metrics caused by solar radiation or cloud. For example, if both GPP and SW peaked at 12 p.m. under normal conditions and both of them peaked at 11 a.m. during the heatwave, then we could not conclude that there was a shift in the GPP diurnal cycle during the heatwave because it was simply caused by the changed time of solar radiation. Therefore, we first determined the peak hour of solar radiation as local noon and then used it as a reference to calculate *C*_GPP_, Hour_peak_, and Ratio_A/M_. As a result, all the diurnal metrics were aligned with the peak time of solar radiation. We also used an alternative approach that involves calculating the difference between *C*_GPP_ and *C*_SW_ to eliminate the effect resulting from radiation (*32*, *67*, *68*).

Figure S4 shows an example of the spatial patterns of *C*_GPP_, Hour_peak_, and Ratio_A/M_. The three metrics consistently reveal contrasting diurnal patterns of GPP between the western and eastern United States. The arid and semiarid regions in the Southwest had higher photosynthesis in the morning, in contrast to the forest and cropland in the eastern United States. We compared the diurnal metrics derived from the gridded GPP and the EC flux data and found that the predicted diurnal metrics were strongly correlated with those based on EC flux data (*R*^2^ = 0.42 to 0.67, *P* < 0.0001; fig. S28), supporting its application at the larger scale regarding the analyses of the impact of the heatwave on the diurnal cycle of GPP. The model better predicted *C*_GPP_ and Ratio_A/M_ than Hour_peak_ because *C*_GPP_ and Ratio_A/M_ were more stable, as they were calculated from multiple hours and less affected by outliers. The model solely based on SW effectively predicted the diurnal metrics for nondrylands but was unable to predict them for drylands.

To examine the effect of heatwaves on the diurnal variations of GPP, we calculated the difference (referred to as “anomaly” henceforth) of these diurnal metrics in the 2020 heatwave period relative to the average in the same period in 2018 and 2019. This approach provided insights into the magnitude of the shift in diurnal GPP metrics attributable to the 2020 heatwave. We recognize, however, that establishing a baseline using mean values from just 2 years may introduce uncertainty. To address this concern and verify the reliability of our findings, we have also incorporated data from 2021 and 2022, thereby extending the baseline period. This additional analysis aids in assessing the consistency and robustness of our results. We then examined how these metrics changed for different vegetation types and aridity conditions with different heat stresses. The vegetation types were determined by the MODIS land cover map, including ENF, evergreen broadleaf forest (EBF), deciduous broadleaf forest (DBF), mixed forest (MF), shrubland, savanna, grassland, and cropland. Because we found that ENF showed shifted diurnal metrics during the heatwave, as did shrubland, savanna, and grassland, while EBF, DBF, MF, and cropland were more resistant to the heat condition, we then discussed them separately as two types based on their responses and combined the three other forest types as non-ENF. The heat stress was quantified by the standardized normalized anomaly of air temperature (Ta_ano) and VPD (VPD_ano) in the 2020 heatwave period relative to the multiyear average during 2000–2019. Daily air temperature and VPD in August from 2000 to 2020 were obtained from the Modern-Era Retrospective analysis for Research and Applications (MERRA-2). The global aridity map was obtained from the Global Aridity Index (Global-Aridity) and the Global Potential Evapo-Transpiration (Global-PET) Geospatial Database (*69*). The AI defined as the ratio of mean annual precipitation to mean annual potential ET ranged from 0 to more than 2 with a higher value indicating a more humid condition. The aridity conditions were grouped by six AI bins (0 to 0.2, 0.2 to 0.4, 0.4 to 0.6, 0.6 to 0.8, 0.8 to 1, and >1) to explore their responses to the heatwave. The AI threshold of 0.65 was used to separate the drylands (AI ≤ 0.65; fig. S2) and nondrylands (AI > 0.65) in our analyses (*45*). We also examined the relationship between *C*_GPP_ and the corresponding change in daily GPP to understand how the shift in diurnal metrics affected the daily aggregated GPP. We further explored at which hour ecosystems had the largest loss of GPP during the heatwave compared to the normal years. This question further sheds light on the symmetrical or asymmetrical diurnal variations of GPP in the heatwave. If the heatwave only inhibits photosynthesis without altering its diurnal shape (i.e., uniformly), then the largest loss of GPP should occur at the time when the vegetation is most productive, such as near noon; otherwise, it should occur at other times.

Last, we further aimed to confirm the robustness of the observed shift in diurnal metrics revealed by gridded GPP estimations using EC flux site data. The site-level validation was twofold: solely based on the EC flux site data and based on modeled GPP data driven by site explanatory variables. During the examined heatwave period (14 to 19 August), not all the sites experienced a severe heatwave, and thus, we extended the heatwave period throughout August. If the site had a positive Ta_ano larger than 1.5 for at least five consecutive days, then it was used to explore the effect of the heatwave. The used sites are listed in table S2. We also selected four sites with different vegetation types to demonstrate how the environmental factors (Ta, VPD, and SW) and GPP varied over the course of a day under normal and heatwave conditions. The four sites included woody savanna (Tonzi Ranch, US-Ton), grassland (Walnut Gulch Kendall Grasslands, US-Wkg), ENF (Valles Caldera Ponderosa Pine, US-Vcp), and cropland (Rosemount I18_South, US-Ro5).

### Exploration of environmental regulation on diurnal cycle of GPP

To further understand the regulation of water and heat stresses on the diurnal behavior of ecosystem photosynthesis, we examined the relationships between three diurnal metrics (*C*_GPP_, Hour_peak_, and Ratio_A/M_) and daily environmental variables for different vegetation types. For the diurnal shift and environmental controls, both results from the regional level based on gridded data and the site level based on EC flux data are provided. For the regional level, the diurnal metrics, VPD, and LST were averaged per year for each vegetation type, and then, their correlations were examined. For the pixel level, we examined their relationship for all the pixels for 3 years and provided the pixel-level relationships for each vegetation type for *C*_GPP_. All the pixels with *C*_GPP_ and Hour_peak_ ranging from 8 to 14 hours are counted to avoid the impact of some outliers on the fitting. The slope of VPD (or LST)–*C*_GPP_ relationship was used to describe the sensitivity of *C*_GPP_ to the variations of VPD and LST. We also carried out analogous analyses to compare the distinct responses between drylands (AI ≤ 0.65) and nondrylands (AI >0.65) and to investigate the effects of varying baseline periods, whether 2 or 4 years. In addition, we also examined the relationships between the three diurnal metrics and environmental variables based on EC flux data with air temperature included in the analyses.

Because we used MODIS NDVI to estimate the hourly GPP estimations, the diurnal asymmetry of GPP if observed was assumed to be mainly resulting from the diurnal variations in LUE, considering generally the symmetrical diurnal cycle of radiation. We then calculated hourly LUE and examined its hourly anomaly during the heatwave relative to normal years to examine how LUE accounted for the shift in diurnal metrics. The strict definition of LUE is GPP divided by the product of photosynthetically active radiation (PAR) and the fraction of absorbed PAR (fPAR). In this study, LUE was approximated by hourly GPP divided by the product of NDVI and hourly SW, considering the following: (i) We only aimed to analyze the relative change of LUE per hour during the heatwave rather than use its absolute value. (ii) NDVI and hourly SW data were already processed and included in our analyses, and NDVI is often used as a proxy of fPAR and SW scales with PAR. We calculated the hourly LUE anomaly (expressed as the difference of LUE under normal and heatwave years divided by LUE in the normal years) to explore its relationship with variations of diurnal metrics. Specifically, we counted for the proportions of pixels with a negative *C*_GPP_ anomaly (morning-shifted) when the decrease of LUE was smaller than 0, 20, and 50%, respectively, and further examined the LUE anomaly across hours for three situations: (i) drylands with Ta_ano ≥ 1, (ii) nondrylands with Ta_ano ≥ 1, and (iii) drylands with Ta_ano ≥ 2. This could indicate how the LUE anomaly changed across hours for different ecosystems and under different heat conditions.

### Calculation of daily GPP and GPP loss during the heatwave

We calculated the daily GPP based on two methods: aggregated from hourly GPP derived from GOES-R (GPP_GOES_) and upscaled from the instantaneous satellite observations (GPP_upscaling_) by assuming an ideal cosine curve and symmetrical diurnal cycle. This temporal upscaling strategy has been widely used to convert instantaneous SIF to daily SIF (*17*, *20*). We followed the temporal upscaling method proposed by (*19*) to obtain daily GPP from hourly GPP. Their main assumption was that the diurnal variation of the energy-related variables (GPP and ET) scaled with that of potential solar radiation (*Rg*_POT_) and the instantaneous GPP or ET could be converted to daily using the ratio of the instantaneous *Rg*_POT_ to the daily total *Rg*_POT_. This method is expected to perform better when there is no substantial asymmetry in environmental conditions between morning and afternoon that alters the diurnal shape of targeted variables. One example of the upscaling method is shown in fig. S29. The predicted GPP from GOES exhibited a highly asymmetrical diurnal cycle with a peak time at 9 a.m., while the upscaled GPP scaled with radiation showed a symmetrical diurnal cycle with a peak time at 12 p.m. The upscaled GPP from GPP at 8 a.m. overestimated the hourly GPP for most hours, leading to an overestimation of 88.2% for the daily GPP, while the upscaled GPP from GPP at 12 p.m. underestimated the hourly GPP for most hours, leading to an underestimation of 60.9% for the daily GPP.

We obtained multiple GPP_upscaling_ values upscaled from five single hours (8 a.m., 10 a.m., 12 p.m., 2 p.m., and 4 p.m.) and then compared their difference with GPP_GOES_ in both normal and heatwave years to provide insight into the effect of selected upscaling time and heat conditions on the resulting daily GPP. The probability distribution of biases was calculated for both drylands and nondrylands for both years. We also estimated the daily GPP based on the fixed hourly LUE upscaling scheme, which assumed that LUE remains invariant over the course of a day, and compared the results with GPP_upscaling_. For example, if we had GPP and SW at 8 a.m., then the LUE at 8 a.m. was used to infer GPP at other times with known hourly SW. The radiation-based upscaling method also implicitly used a fixed hourly LUE throughout the day.

We illustrated how the regional-averaged GPP_upscaling_ under/overestimated the GPP_GOES_ across hours in both normal and heatwave years and how this bias changed with increasing heat conditions using GPP_upscaling_ derived from 2 p.m. as an example. The bias was calculated as the difference of GPP_upscaling_ and GPP_GOES_ divided by GPP_GOES_ and then compared across hours in both years. The difference of bias between the normal and heatwave years (bias_normal_ − bias_heatwave_) was then calculated for each hour under four conditions: (i) Ta_ano ≥ 1, (ii) Ta_ano ≥ 2, (iii) Ta_ano ≥ 2 and only for shrubland and grassland, and (iv) Ta_ano ≥ 3 and only for shrubland and grassland. The heat conditions and diurnal asymmetry in GPP increase from conditions 1 to 4. We further examined how the bias was propagated to the calculation of GPP loss across hours during the heatwave. We first calculated the GPP loss in percentage based on both the GPP_upscaling_ (the difference of GPP_upscaling_heatwave_ and GPP_upscaling_normal_ divided by GPP_upscaling_normal_) and GPP_GOES_ (the difference of GPP_GOES_heatwave_ and GPP_GOES_normal_ divided by GPP_GOES _normal_) for each hour and then examined how this difference by two methods varied under the four aforementioned conditions. Last, on the basis of the daily GPP estimates from two methods, we calculated the regional total GPP from 14 to 19 August in the normal and heatwave years and compared their difference in quantifying the total dryland GPP loss during the heatwave across the region (Ta_ano ≥ 1 and AI ≤ 0.65). For the upscaling methods, we provided all the results every two hours based on GPP_upscaling_ derived from 8 a.m. to 4 p.m., respectively.
