# Supplementary material for: New-generation geostationary satellite reveals widespread midday depression in dryland photosynthesis during 2020 western U.S. heatwave
Source: Sci Adv. 2023 Aug 2;9(31):eadi0775. doi: 10.1126/sciadv.adi0775 (PMC10396307; doi:10.1126/sciadv.adi0775)
Supplement: Supplementary file 1 — Figs. S1 to S29 Tables S1 to S6 [file sciadv.adi0775_sm.pdf]

Science Advances
AAAS

Supplementary Materials for

New-generation geostationary satellite reveals widespread midday depression in dryland photosynthesis during 2020 western U.S. heatwave

Xing Li *et al.*

Corresponding author: Youngryel Ryu, ryuyr77@gmail.com

Sci. Adv. **9**, eadi0775 (2023)
DOI: 10.1126/sciadv.adi0775

This PDF file includes:

Figs. S1 to S29
Tables S1 to S6

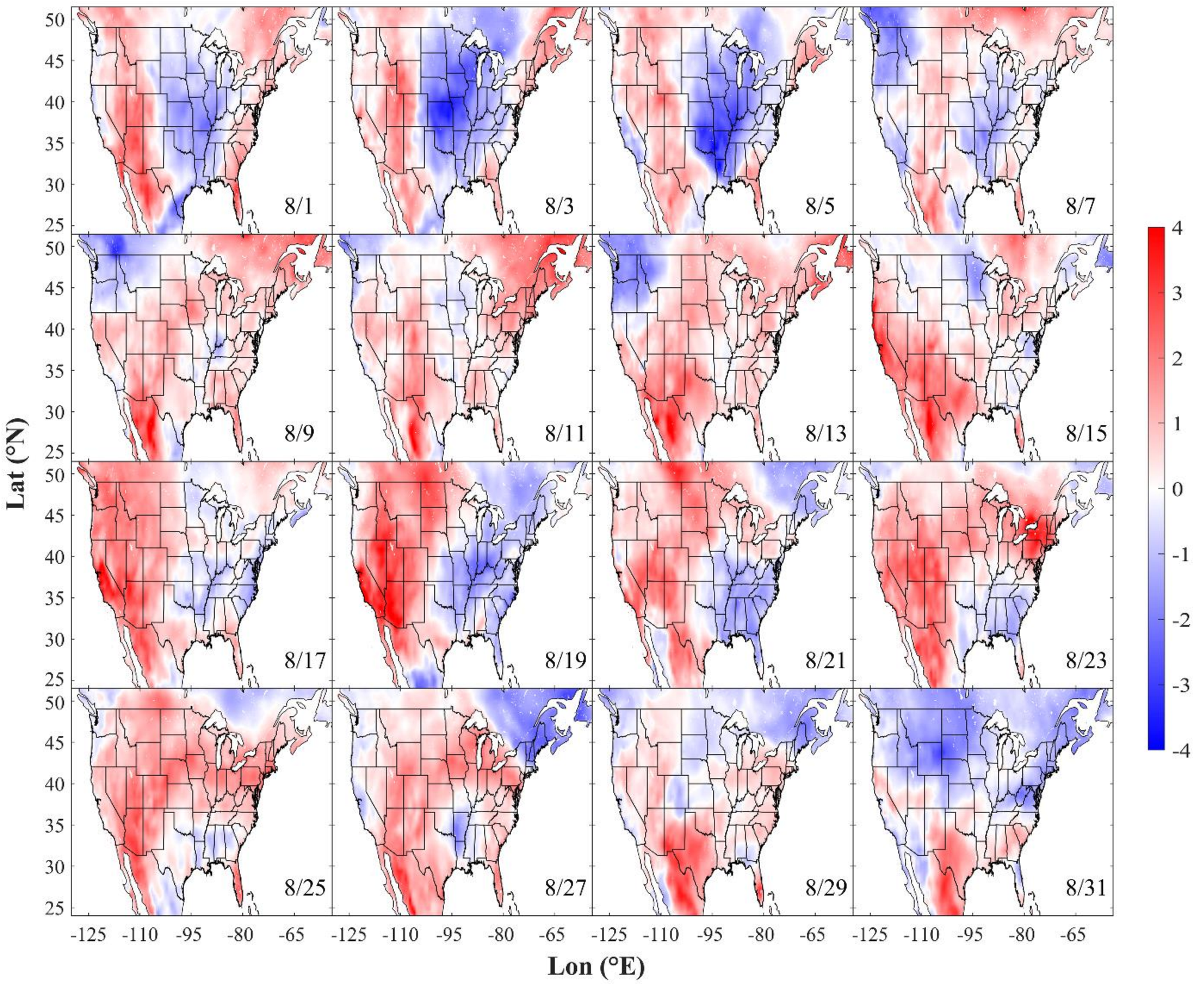

Fig. S1. Evolution of heat condition (standardized normalized anomaly of air temperature from MERRA-2) every two days throughout August 2020. The red/blue pixels indicate higher/lower air temperature in 2020 relative to multiyear mean temperature during 2000–2019.

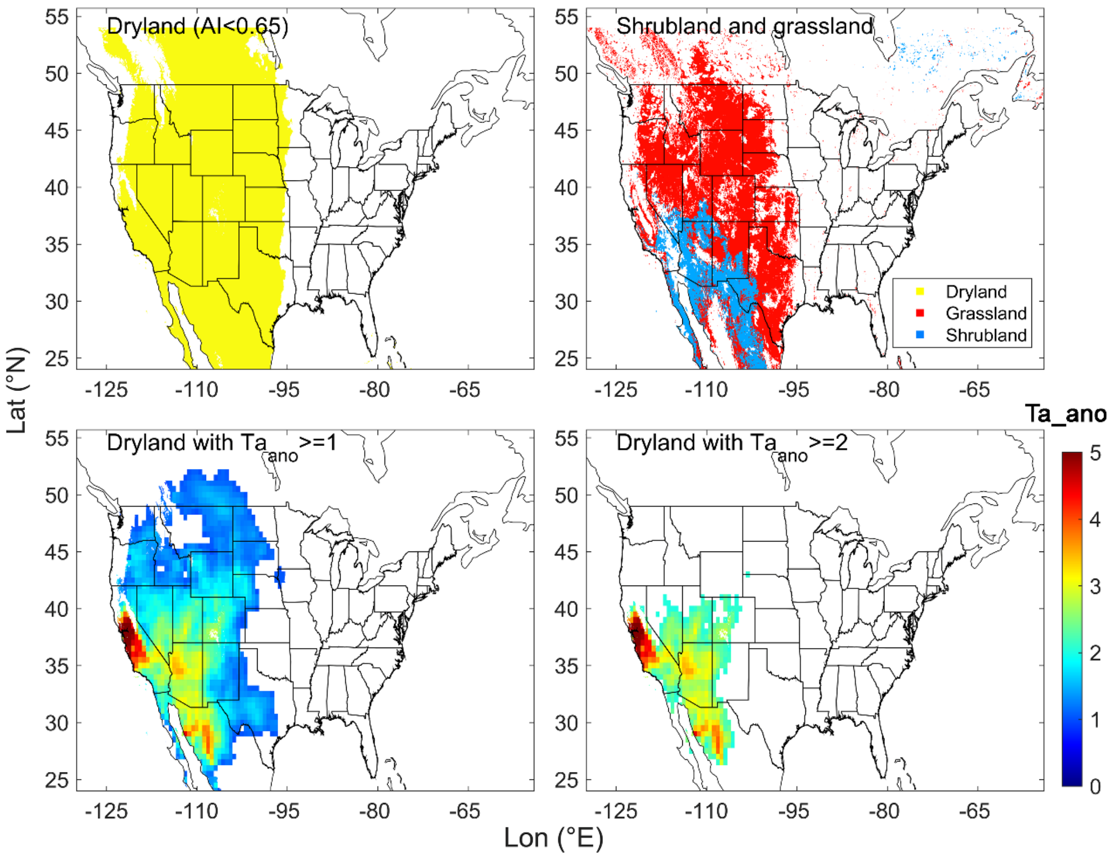

Fig. S2. Geographic distribution of dryland, shrubland, and grassland across the CONUS. The bottom panel shows the areas of dryland subjected to standardized normalized anomaly of air temperature greater than 1 (left) and 2 (right). The legend at the bottom right shows the details of temperature anomaly from 0 to 5. Dryland regions are delineated based on an aridity index (AI) of 0.65 or less.

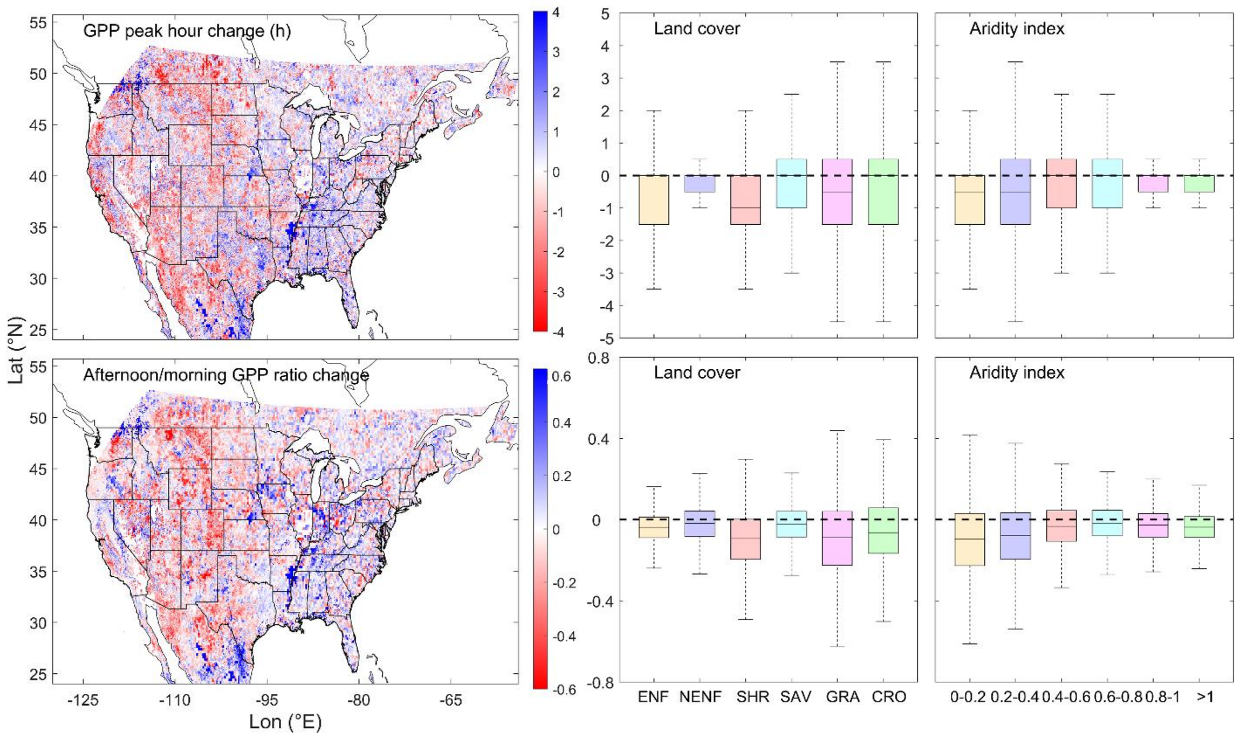

Fig. S3. Change of GPP peak hour and afternoon GPP/morning GPP ratio during the heatwave from August 14 to 19, 2020 relative to 2018 and 2019 across the CONUS. The middle and right columns show different responses of diurnal metrics to the heatwave across vegetation types and along aridity gradients (smaller AI values indicate more arid), respectively. ENF, NENF (or Non-ENF), SHR, SAV, GRA, and CRO represent evergreen needleleaf forest, other forests expect for ENF, shrubland, savanna, grassland, and cropland, respectively. Boxplots illustrate the distribution of diurnal change: the box represents the interquartile range (IQR), containing data from the 25th percentile (Q1) to the 75th percentile (Q3); the horizontal line inside the box indicates the median (50th percentile); the whiskers extend to the minimum and maximum values within 1.5 times the IQR from Q1 and Q3, respectively; the outliers beyond this range are plotted as individual red plus symbols.

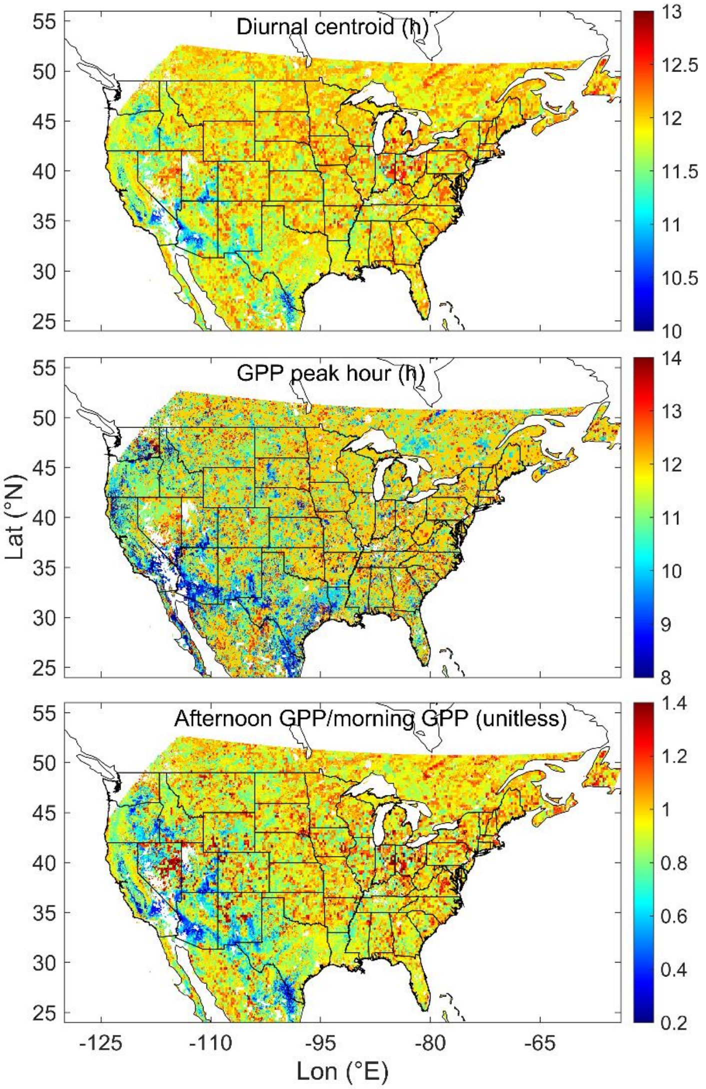

Fig. S4. Example of regional diurnal metric maps (C_{GPP}, $Hour_{peak}$, and $Ratio_{A/M}$) during August 14 to 19 in the normal year 2018 across the CONUS.

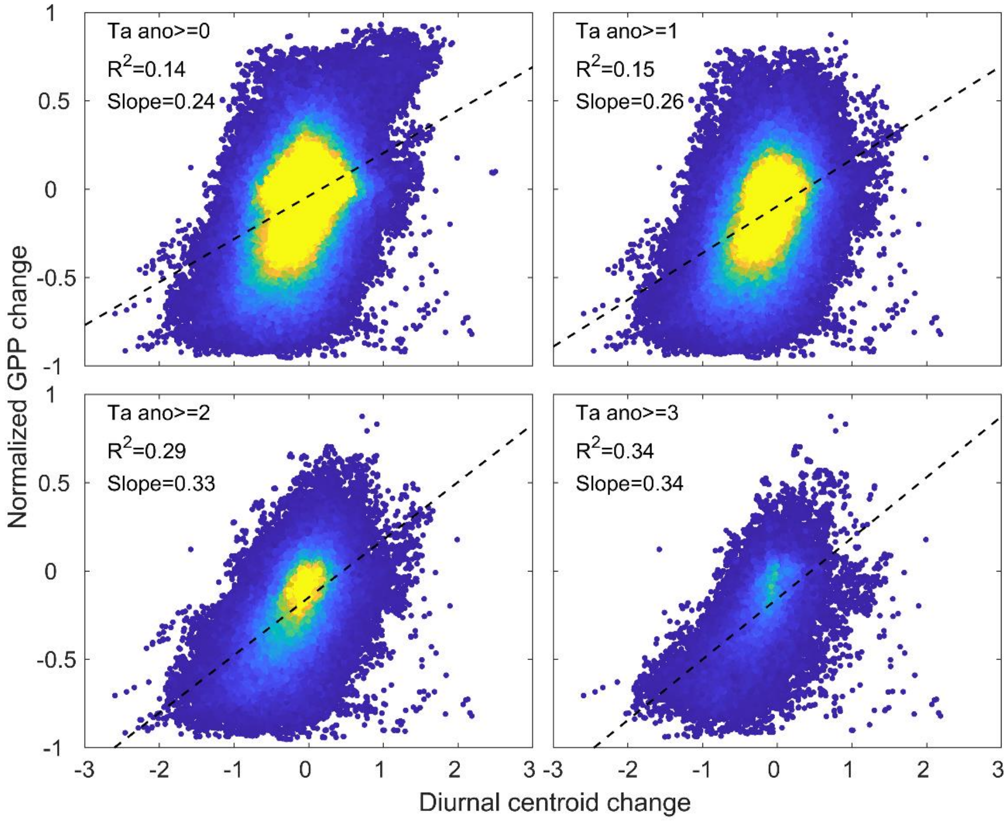

Fig. S5. The relationship between diurnal centroid change and normalized daily GPP change under different heat conditions grouped by air temperature (Ta) anomaly. The normalized daily GPP change was calculated as the difference of GPP in heatwave and normal years divided their sum. Ta anomaly > 0 indicates that all the pixels having Ta anomaly larger than 0 were included.

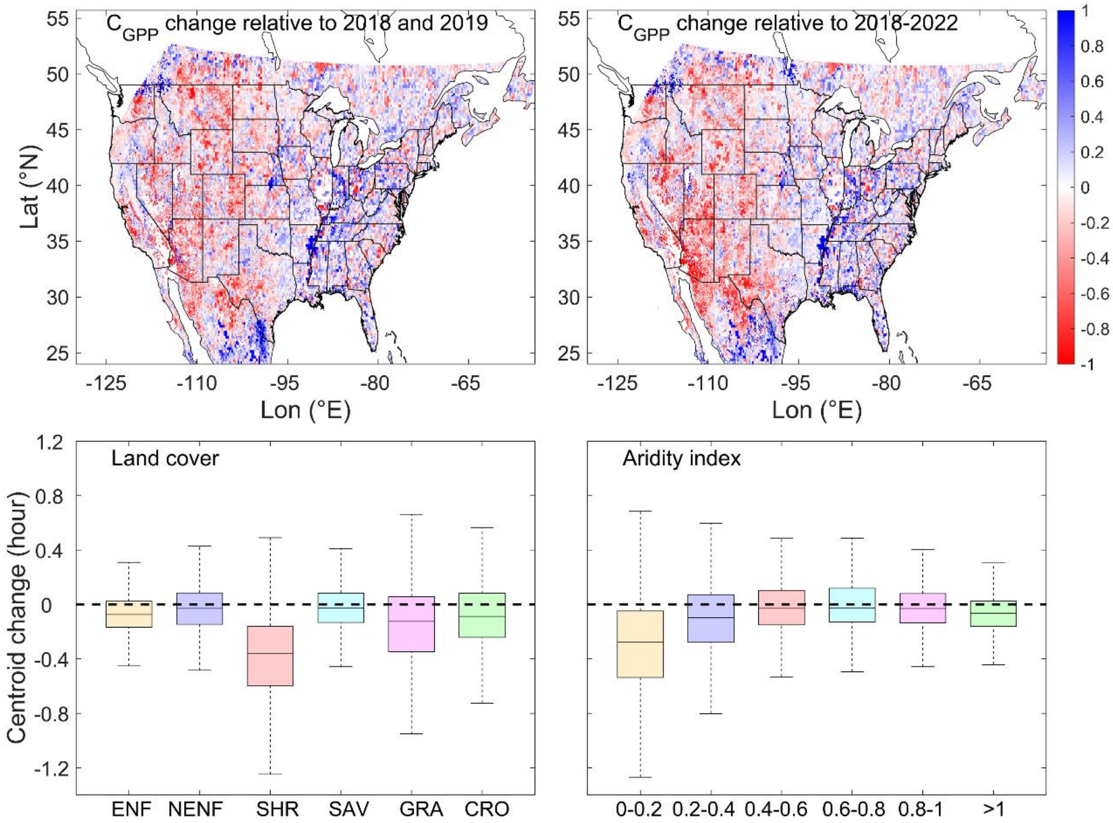

Fig. S6. Change in diurnal centroid of GPP (C_{GPP}) during the heatwave from August 14 to 19, 2020 relative to reference years across the CONUS. Upper panel shows C_{GPP} change in 2020 relative to two-year average (left: 2018 and 2019) and four-year average (right: 2018, 2019, 2021, and 2022), respectively; the lower panel shows different responses of diurnal metrics to the heatwave across vegetation types and along aridity gradients based on four-year baseline. The definitions of boxplot elements are the same as those in Fig. S3. The results based on four-year baseline are highly consistent with those based on two-year baseline (Fig. 1).

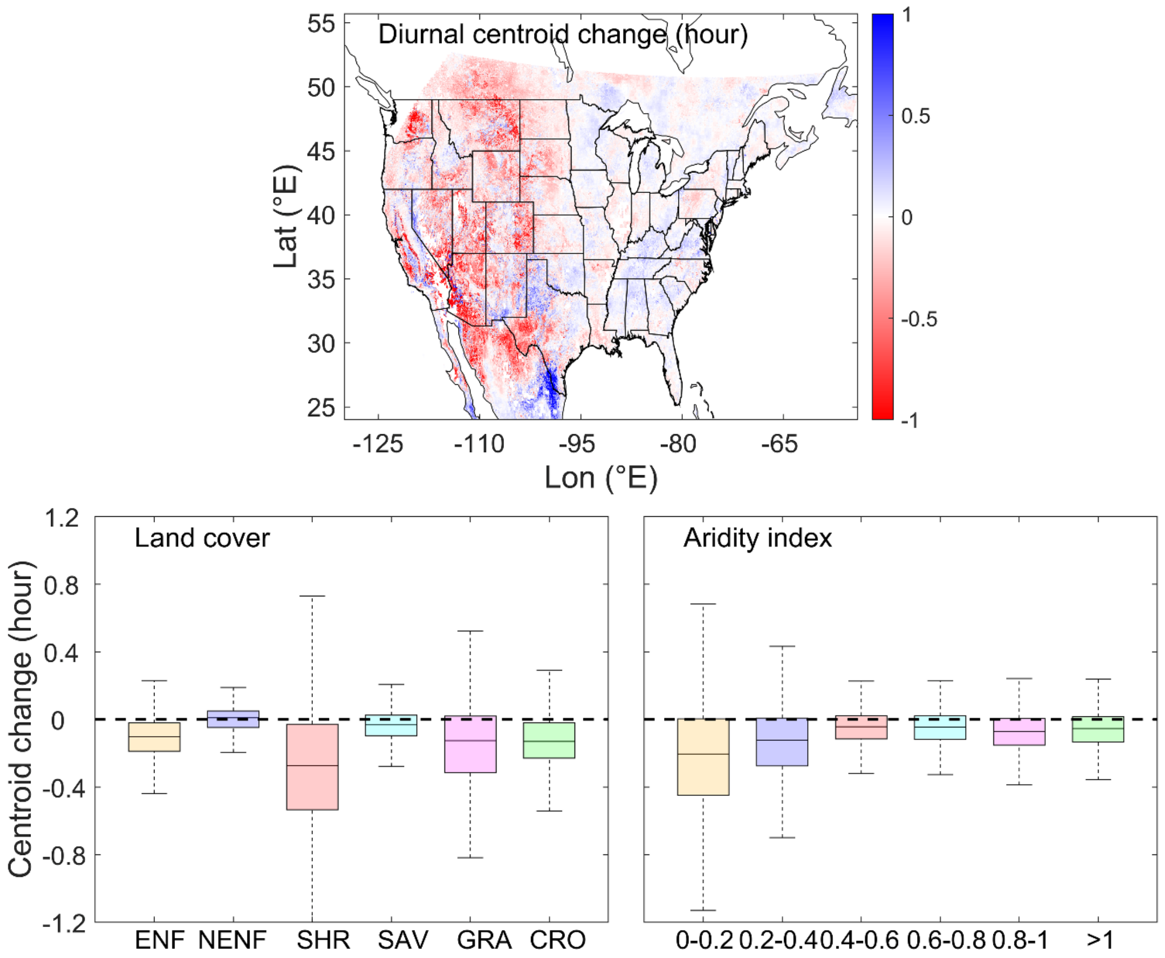

Fig. S7. Change in diurnal centroid of GPP (C_{GPP}) during the heatwave from August 14 to 19, 2020 relative to reference years across the CONUS based on an alternative approach to eliminate the effect resulting from radiation. Upper panel shows C_{GPP} change in 2020 relative to two-year average (2018 and 2019); the lower panel shows different responses of diurnal metrics to the heatwave across vegetation types and along aridity gradients. The definitions of boxplot elements are the same as those in Fig. S3.

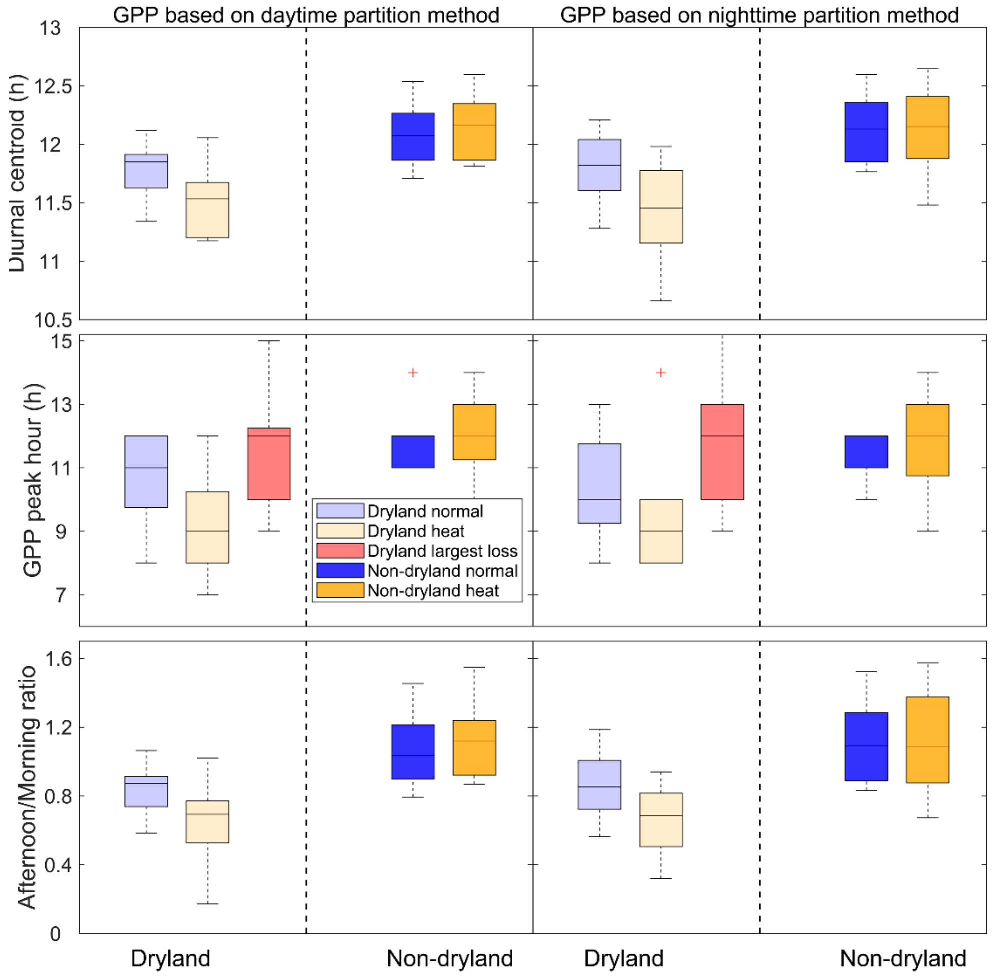

Fig. S8. The diurnal shift of dryland GPP revealed by site-level data: change of three diurnal metrics (diurnal centroid, GPP peak hour or afternoon/morning GPP ratio) during the heatwave relative to two reference years (i.e., 2018 and 2019). The definitions of boxplot elements are the same as those in Fig. S3. Both results based on GPP from daytime and nighttime partitioning methods are provided. The examined sites are marked with asterisk in Table S2. Since Fig. 1 shows the contrasting responses of ENF to the heatwave with other forests, the ENF was included in drylands not forests simply due to their similar responses.

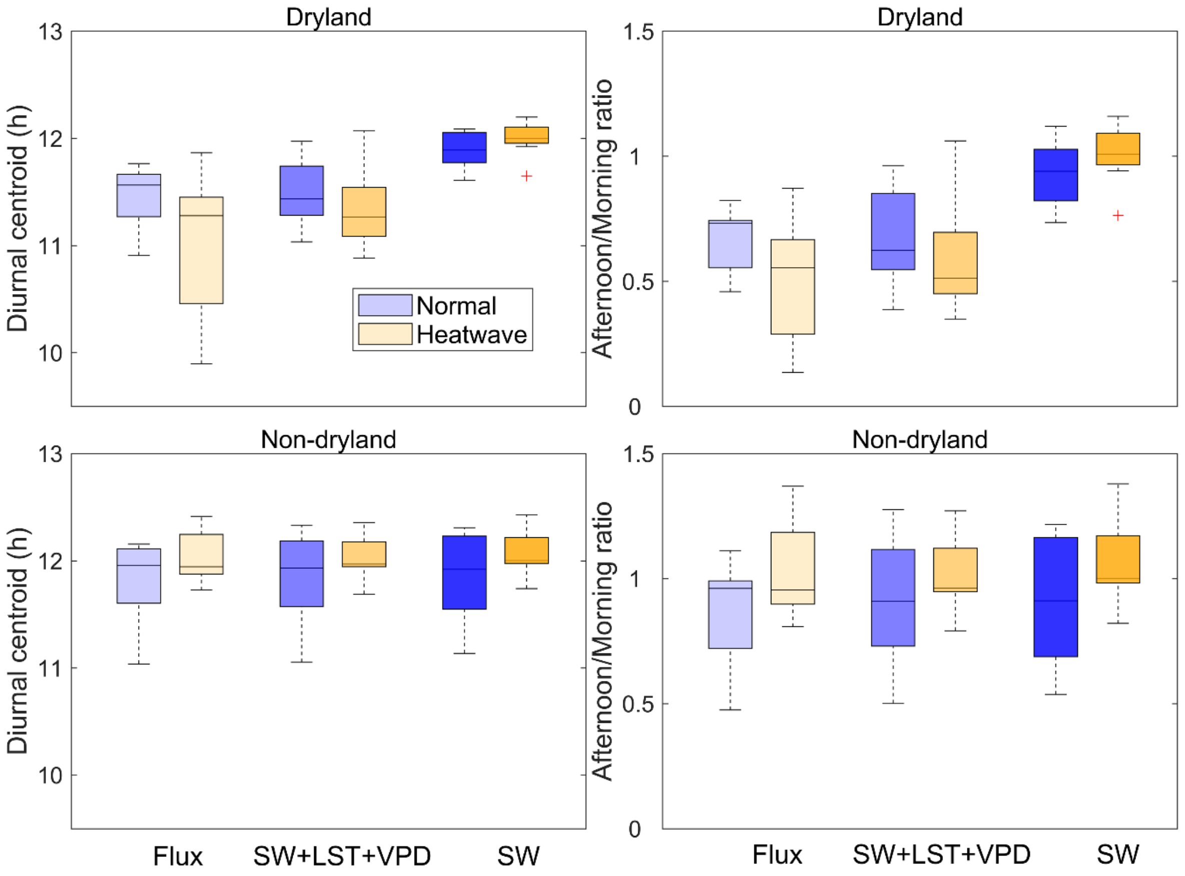

Fig. S9. The diurnal shift of dryland GPP revealed by modelled GPP at the site level: change of diurnal centroid and afternoon/morning GPP ratio during the heatwave relative to two reference years (i.e., 2018 and 2019). Different with Fig. S8, this result was derived from the Cubist model driven by site-level data. The definitions of boxplot elements are the same as those in Fig. S3.

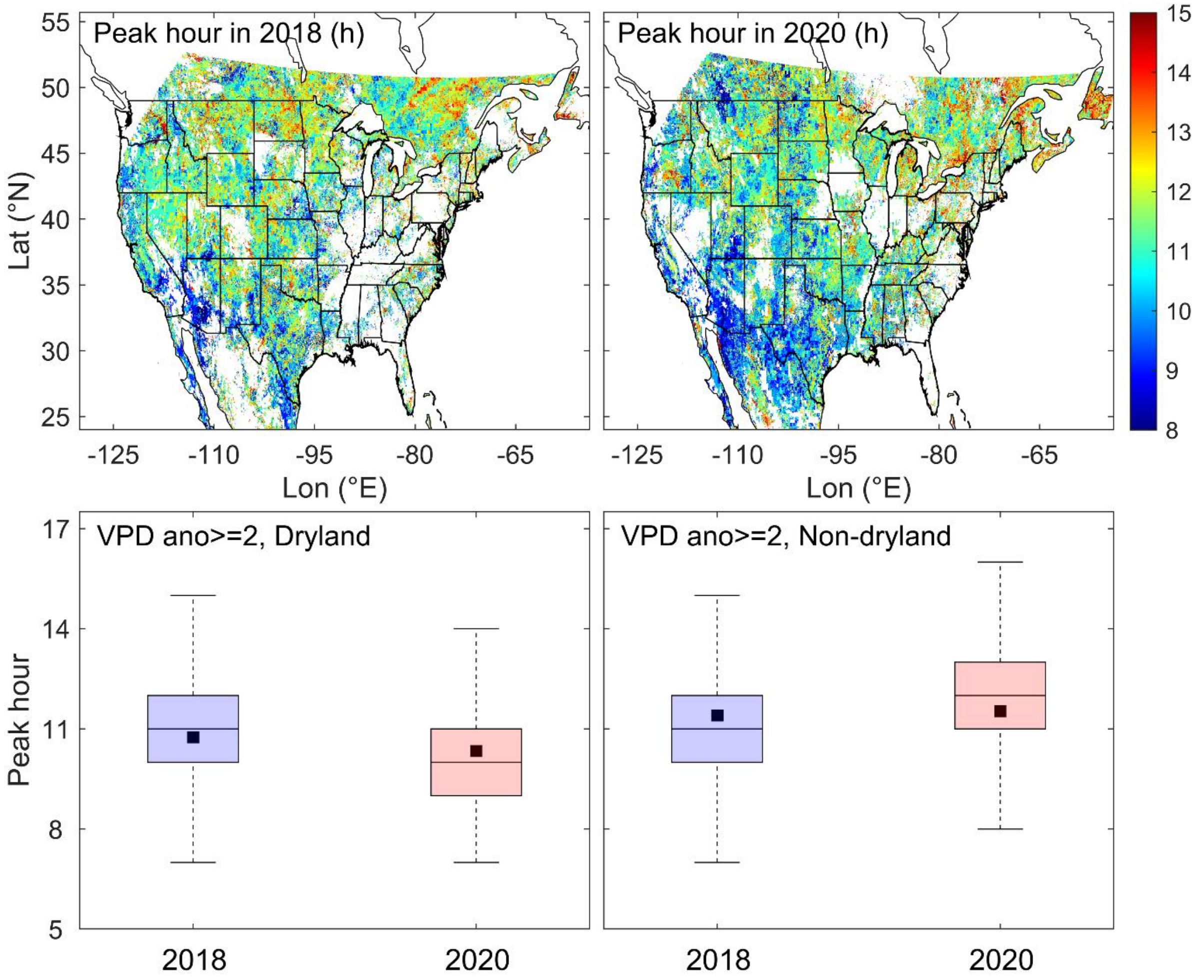

Fig. S10. The diurnal shift of dryland GPP during the heatwave revealed using original GOES LST data without gap-filling. VPD ano >= 2 indicates that VPD anomaly during the heatwave is larger than 2. The definitions of boxplot elements are the same as those in Fig. S3 with filled square symbol representing the mean value.

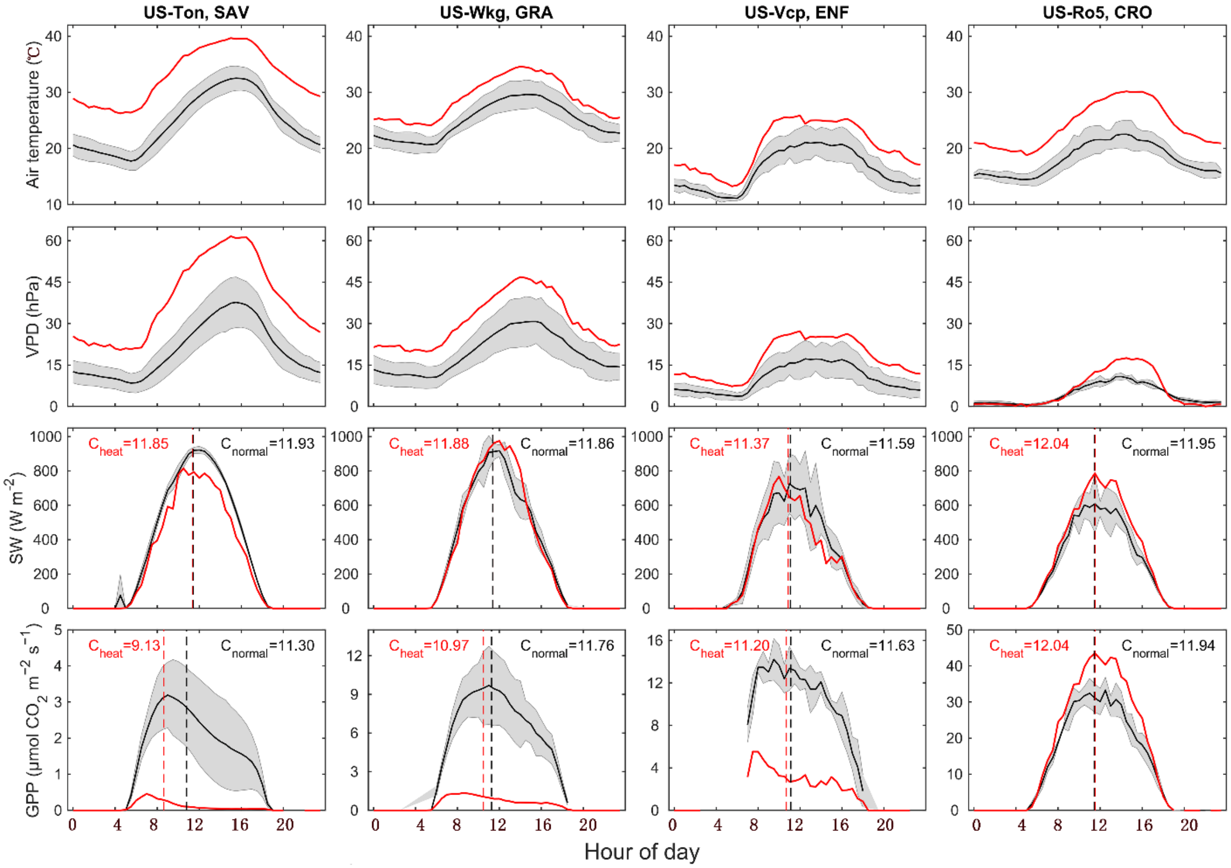

Fig. S11. The diurnal cycles of three environmental variables (air temperature, vapor pressure deficit, incoming shortwave radiation) and GPP under normal and heatwave conditions for four EC flux sites. The red curves indicate the hourly variables during the heatwave, and black curves indicate the hourly variables in the normal years. The vertical dotted lines represent the diurnal centroid of GPP. The examined four sites include Tonzi Ranch (US-Ton, woody savanna), Walnut Gulch Kendall Grasslands (US-Wkg, grassland), Valles Caldera Ponderosa Pine (US-Vcp, ENF), and Rosemount I18_South (US-Ro5, cropland). The hours mentioned here correspond to local time.

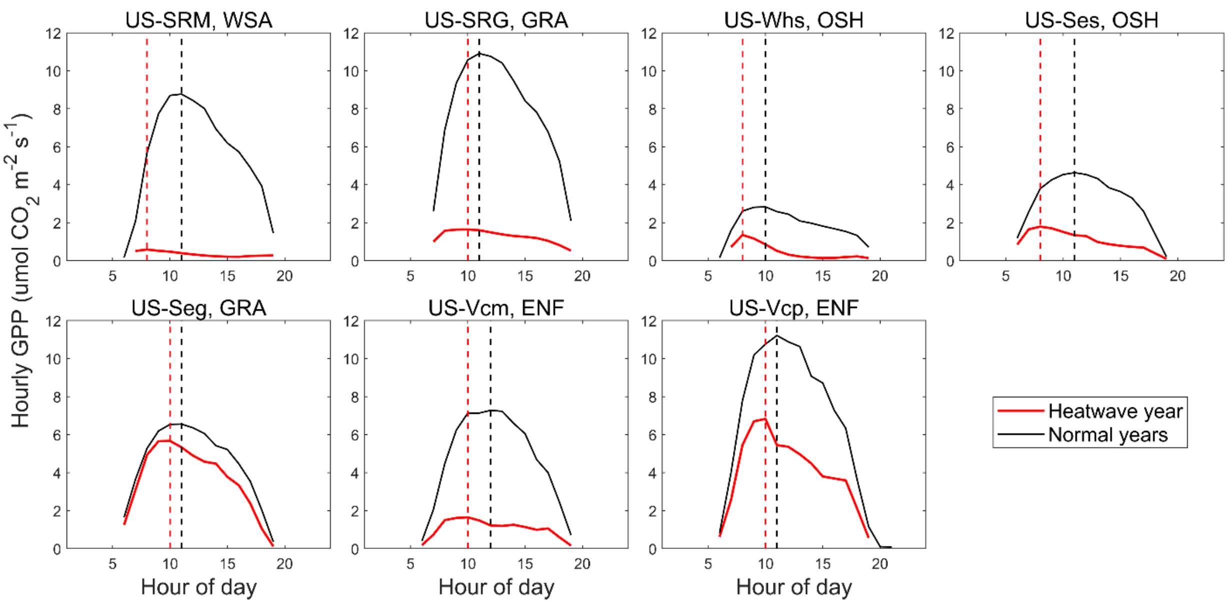

Fig. S12. The diurnal cycles of GPP under normal and heatwave conditions for another seven dryland sites. The red curves indicate the hourly GPP during the heatwave, and black curves indicate the hourly GPP in the normal years. The vertical dotted lines represent the peak hour of GPP. The hours mentioned here correspond to local time.

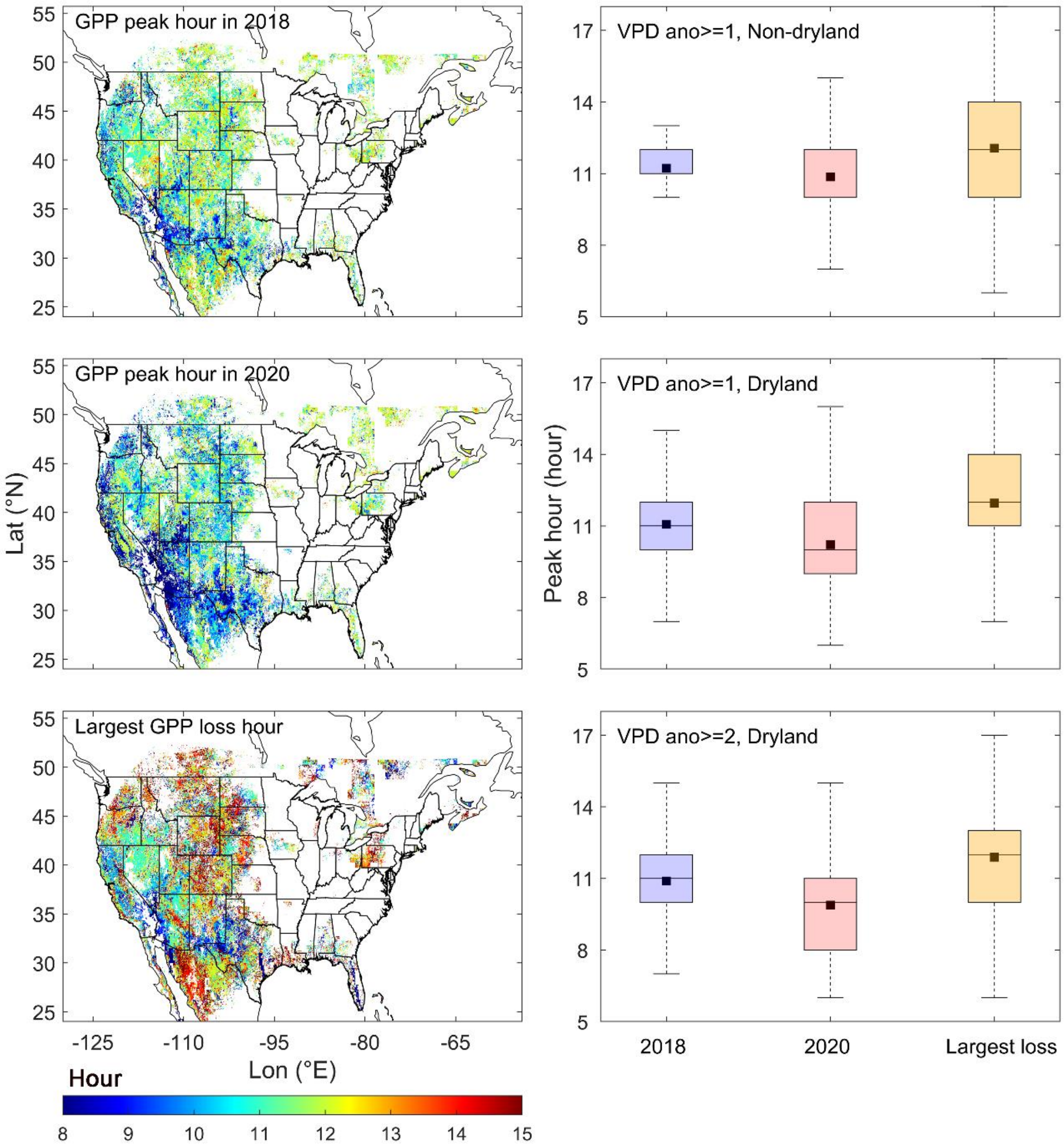

Fig. S13. GPP peak time in 2018 and 2020 and the largest GPP loss hour during the heatwave. The right column shows three examples for drylands or non-drylands under different VPD conditions. VPD ano >=1 or 2 indicates that VPD anomaly during the heatwave is larger than 1 or 2. The definitions of boxplot elements are the same as those in Fig. S3 with filled square symbol representing the mean value.

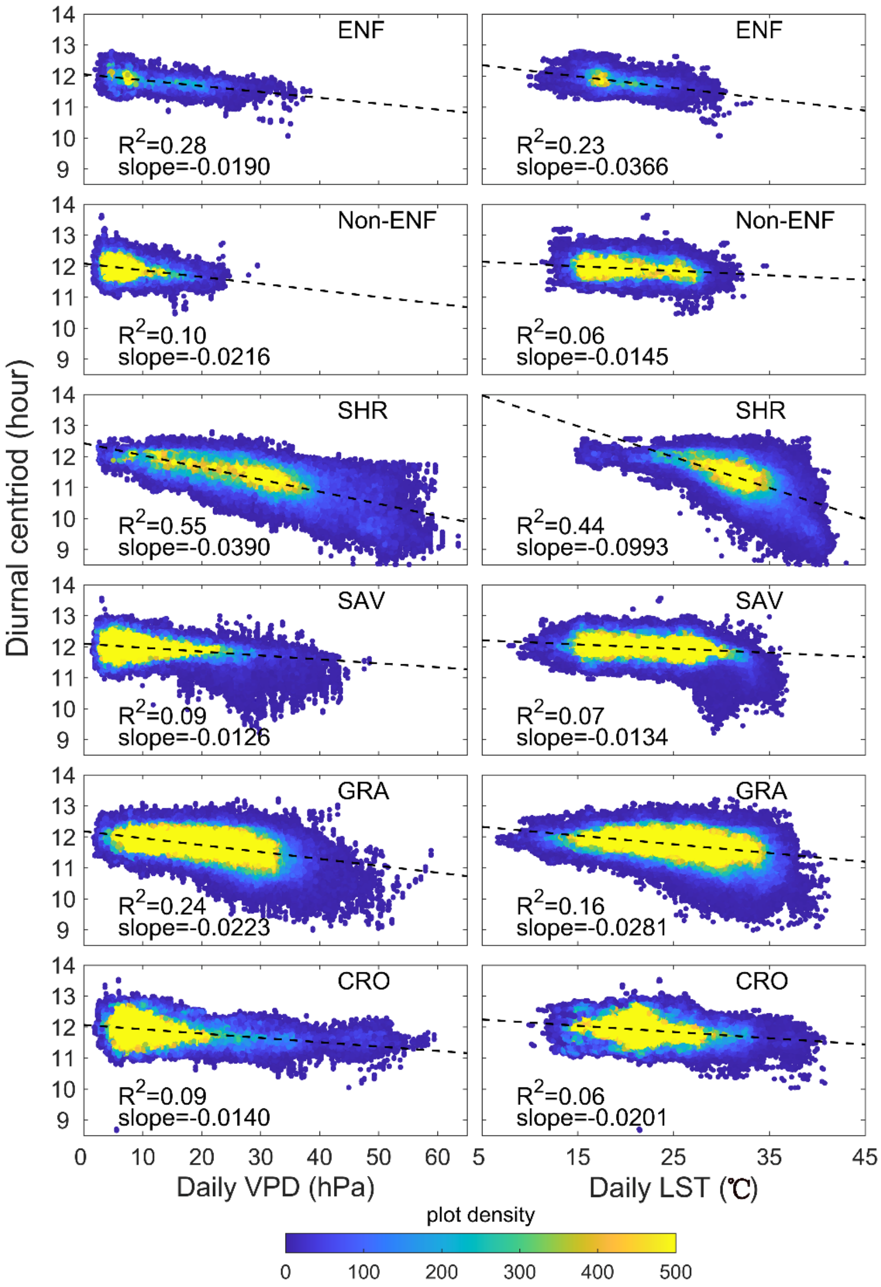

Fig. S14. The relationship between diurnal centroid (C_{GPP}) and daily VPD and LST for different vegetation types at the pixel level.

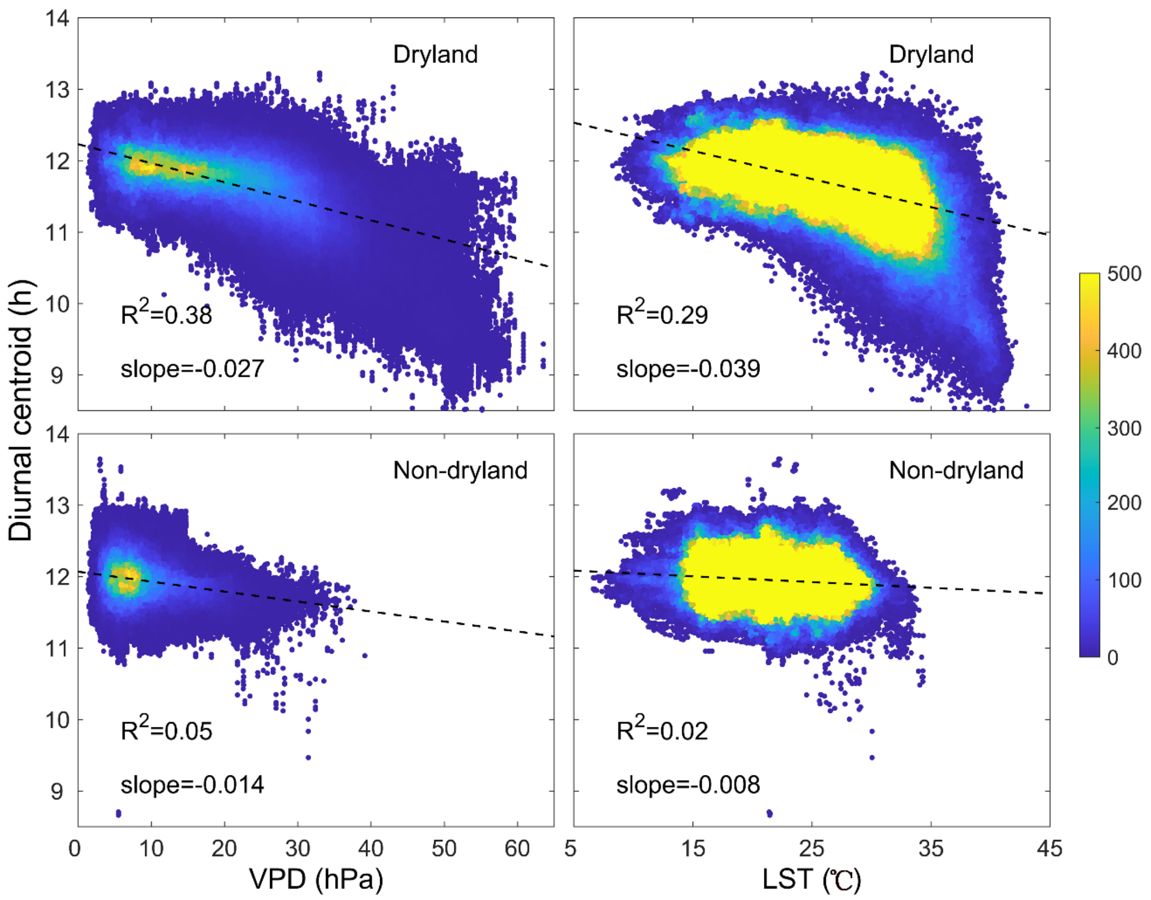

Fig. S15. The relationship between diurnal centroid (C_{GPP}) and daily VPD and LST for drylands and non-drylands at the pixel level.

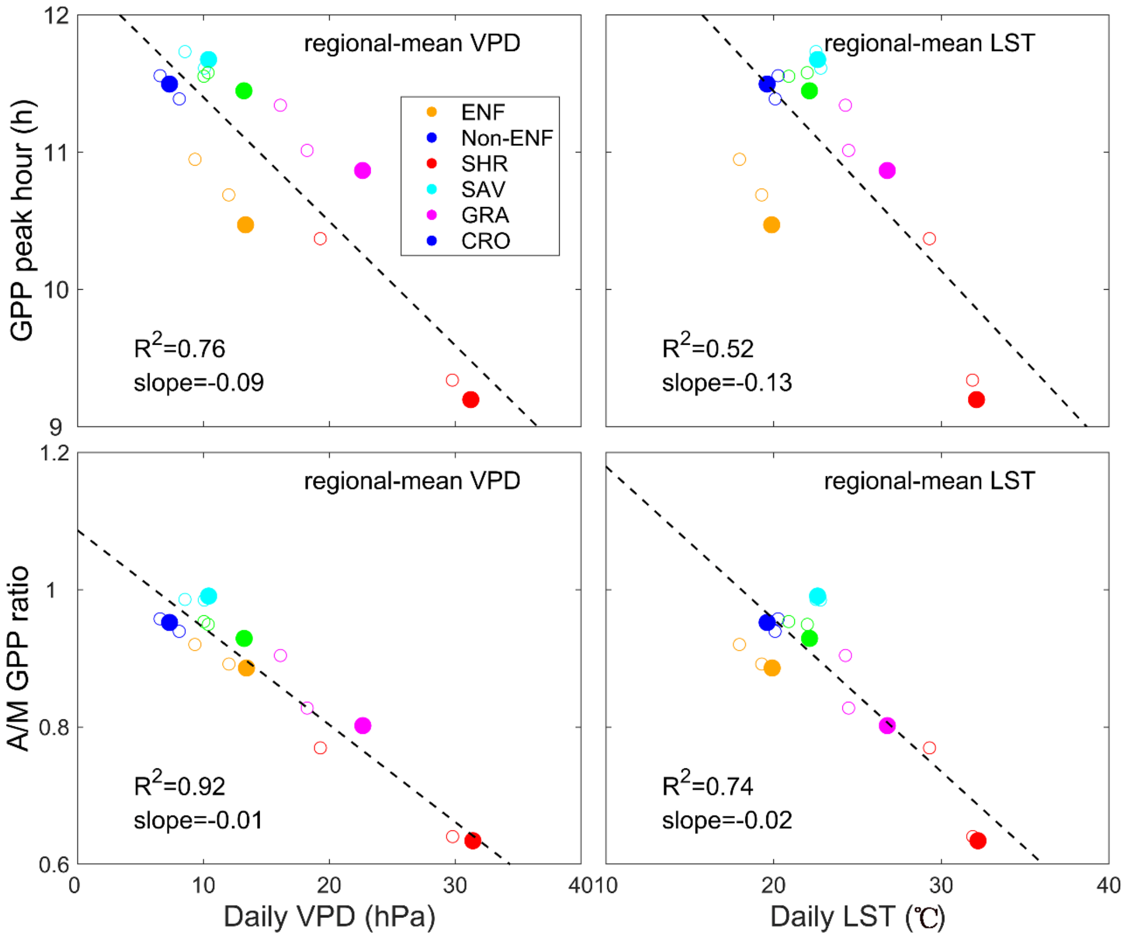

Fig. S16. The relationship between GPP peak hour (or ratio of afternoon GPP to morning GPP) and daily VPD (or LST) for different vegetation types at the regional level.

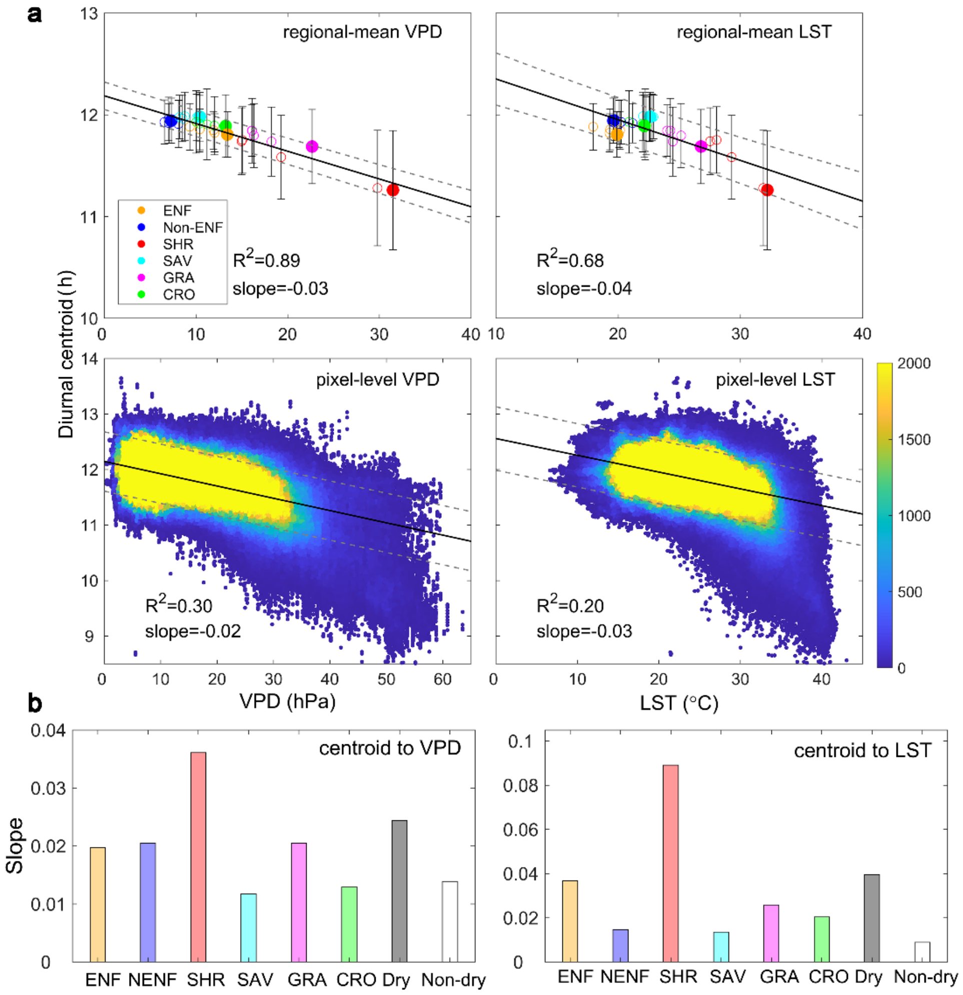

Fig. S17. Relationship between diurnal centroid (C_{GPP}) and daily VPD (or LST) based on four-year baseline. a. Regional-mean and pixel-level relationships between C_{GPP} and VPD (or LST). Filled circles represent the heatwave year, and hollow circles represent normal years with error bars indicating the standard deviation of C_{GPP}. The solid line represents the best-fit line derived from linear regression analysis, and two dashed lines represent the 95% confidence interval for the regression estimate. There are 30 circles in the first row (five years multiplied by six vegetation types). b. Illustrates the slope of VPD (or LST) - C_{GPP} linear relationship for different vegetation types and for drylands or non-drylands. NENF (or Non-ENF) represents other forests, excluding ENF. The units of slope are hour per hPa and hour per °C, respectively.

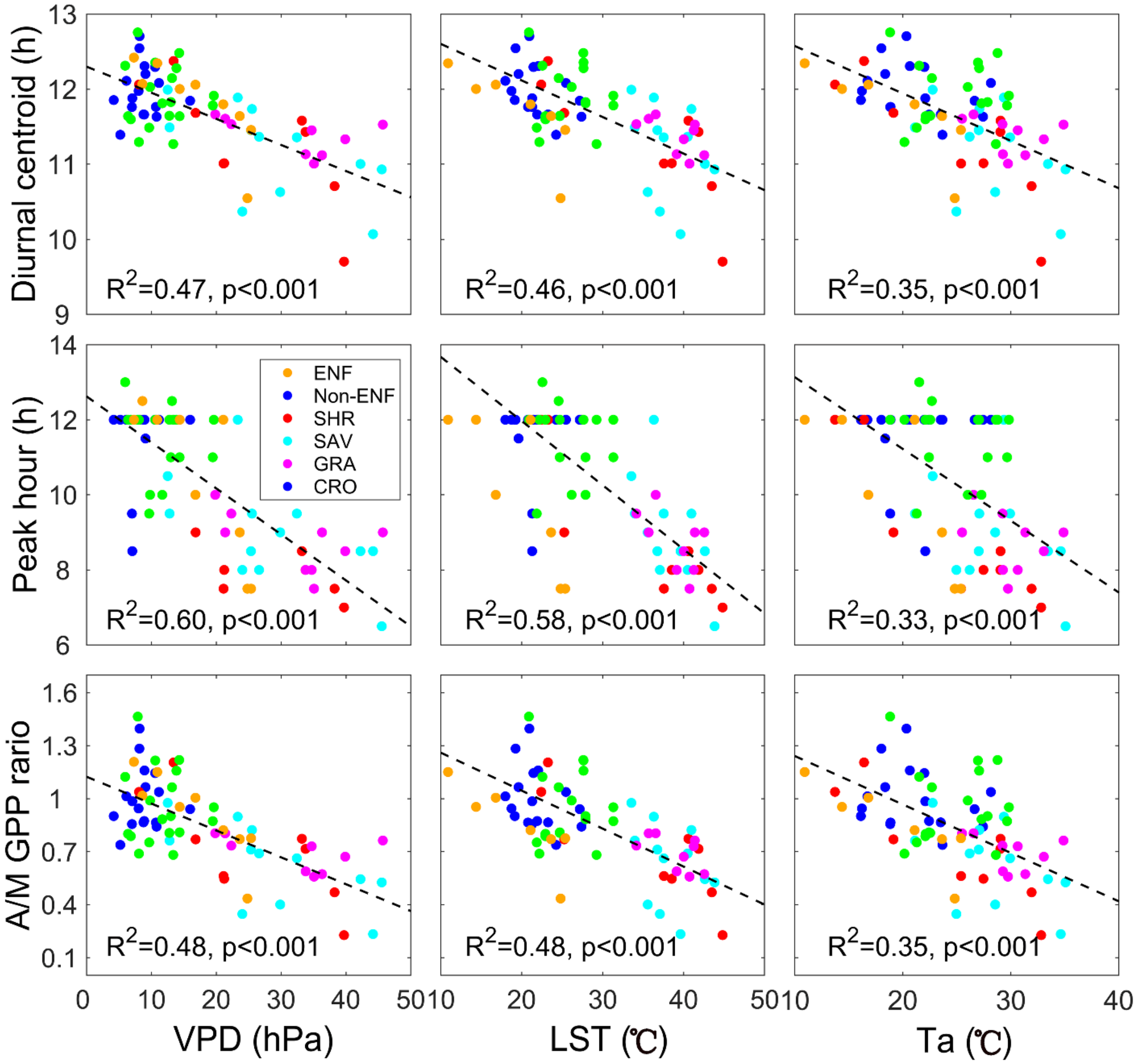

Fig. S18. The relationship between three diurnal metrics (diurnal centroid, peak hour, afternoon/morning GPP ratio) and three environmental variables (VPD, LST, Ta). Each color represents one of six vegetation types.

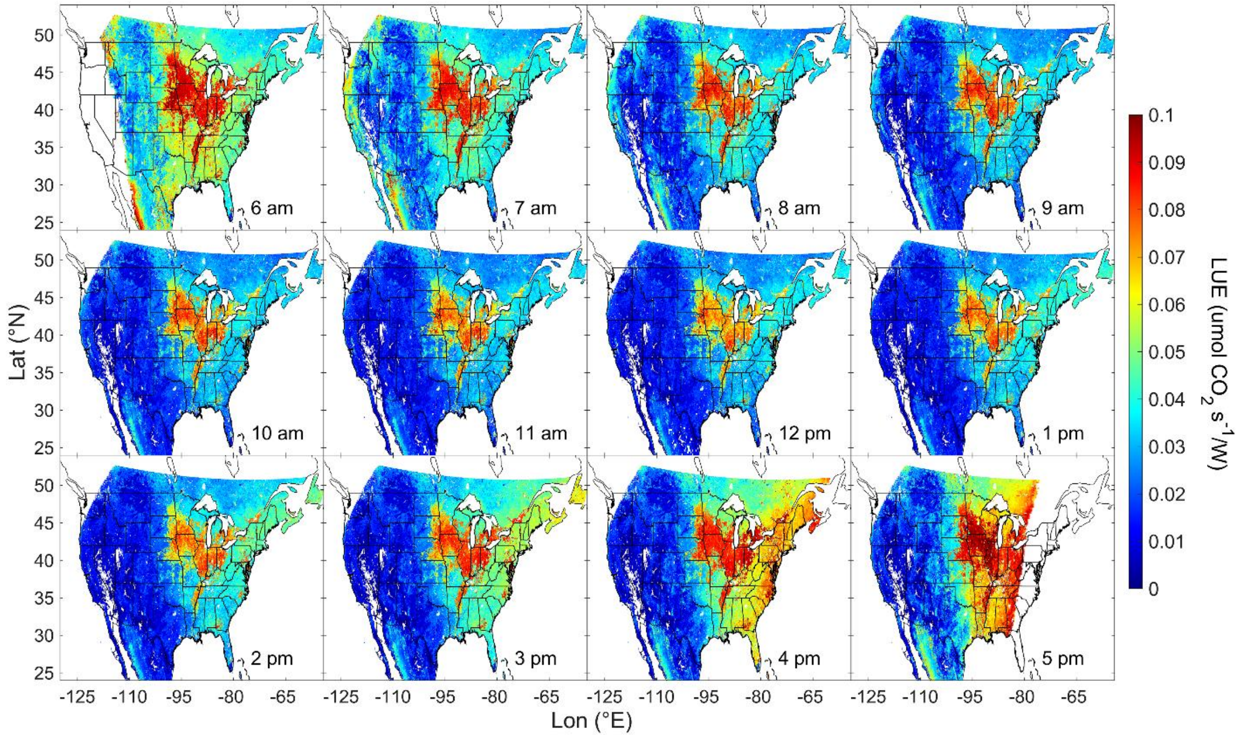

Fig. S19. Example of regional hourly LUE across the CONUS from 6 am to 5 pm on August 1, 2018. The hours shown here are indicated by Pacific Daylight Time (PDT) which is 7 hours behind Coordinated Universal Time (UTC).

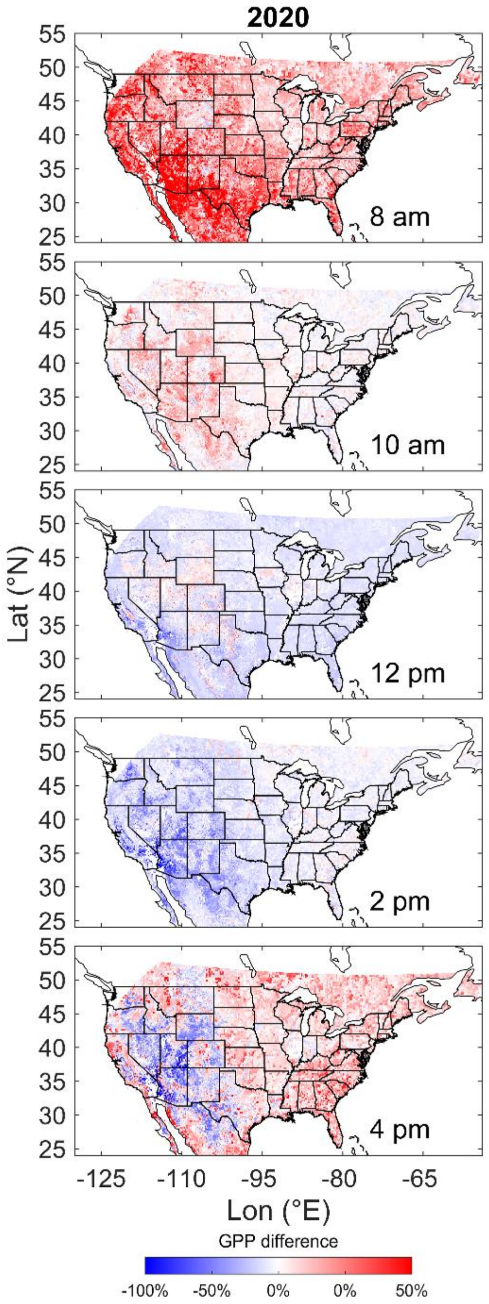

Fig. S20. The difference of daily GPP aggregated from hourly GPP based on GOES-R and based on upscaling from fixed hourly LUE during August 14–19 in the heatwave year. The difference is calculated as ($GPP_{fixedLUE}$-GPP_{GOES})/GPP_{GOES}.

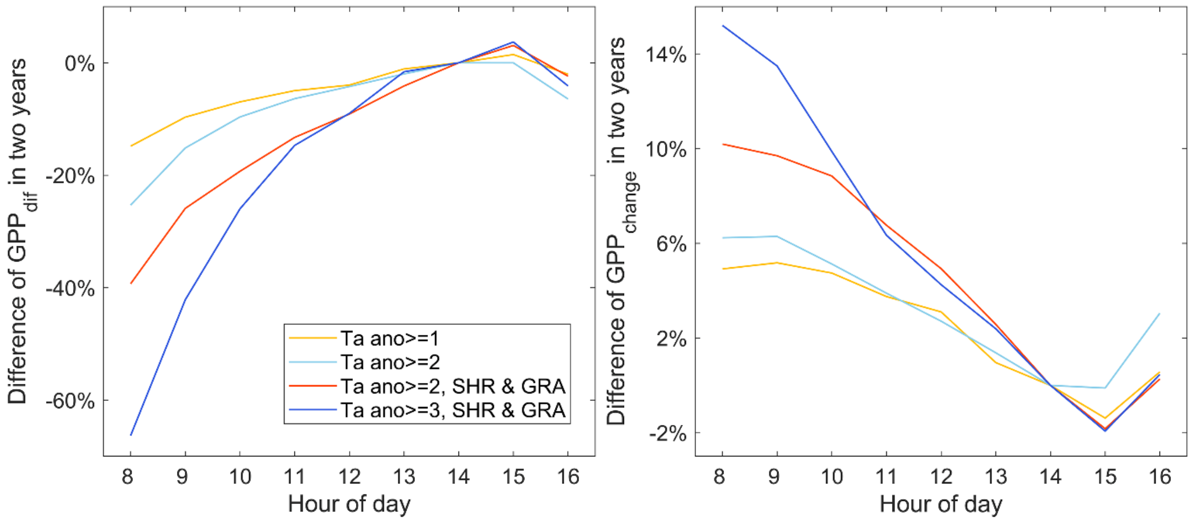

Fig. S21. Difference of $GPP_{upscaling}$ and GPP_{GOES} between the normal and heatwave years (i.e., difference of $GPP_{upscaling}$ and GPP_{GOES} in the normal year minus difference of them in the heatwave year, left) and biases on GPP loss (i.e., loss percentage in GPP based on $GPP_{upscaling}$ minus loss percentage in GPP based on GPP_{GOES}, right) change under four conditions: 1) Ta_ano >= 1; 2) Ta_ano >= 2; 3) Ta_ano >= 2 and only for shrubland and grassland; and 4) Ta_ano >= 3 and only for shrubland and grassland. Heat conditions and diurnal asymmetry in GPP increase from condition 1 to 4. For example, the difference of $GPP_{upscaling}$ and GPP_{GOES} at 8 am between two years is only about -15% (yellow curve on the left) under condition 1, but increases as high as ~70% under condition 4 (blue curve on the left). Similarly, difference in GPP loss between two methods is only 5% (yellow curve on the right) under condition 1 but increases to 15% under condition 4 (blue curve on the right). The hours mentioned here correspond to local time.

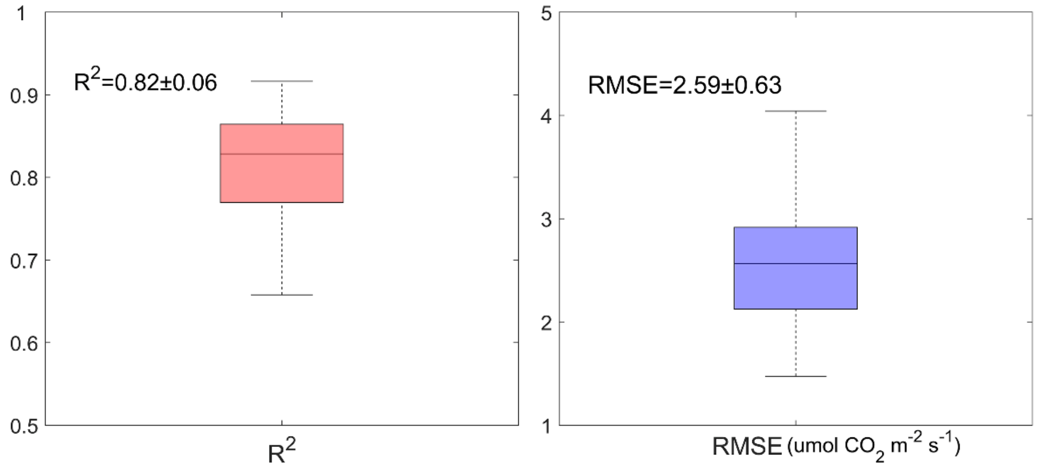

Fig. S22. Evaluation of Cubist model in estimating the hourly GPP: R^2 (left) and RMSE (right). Here the data from the same sites were not used for training, and the training and validation processes were randomly repeated for 200 times.

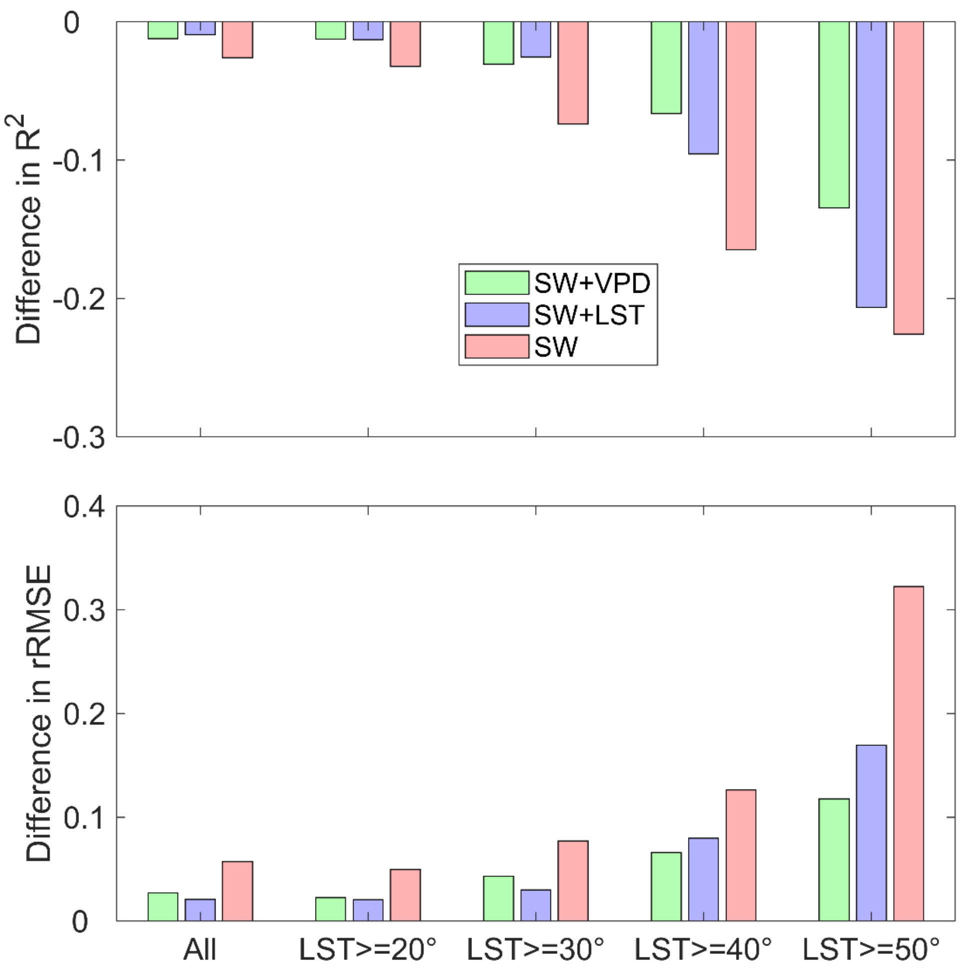

Fig. S23. The difference of R^2 and rRMSE between the three other tested Cubist models and our selected model for estimating hourly GPP under different land surface temperature (LST) conditions. The selected model includes three environmental variables: SW+LST+VPD, and the tested three models exclude either VPD or LST or both of them. 'All' indicates that the samples include all the LST conditions. For example, for samples with LST >= 40 ℃, models with SW plus either VPD or LST showed 0.07 and 0.10 of decrease in R^2, and 0.07 and 0.08 of increase in rRMSE, respectively, while the model only with SW had worst performance with a 0.16 decrease in R^2 and a 0.13 increase in rRMSE.

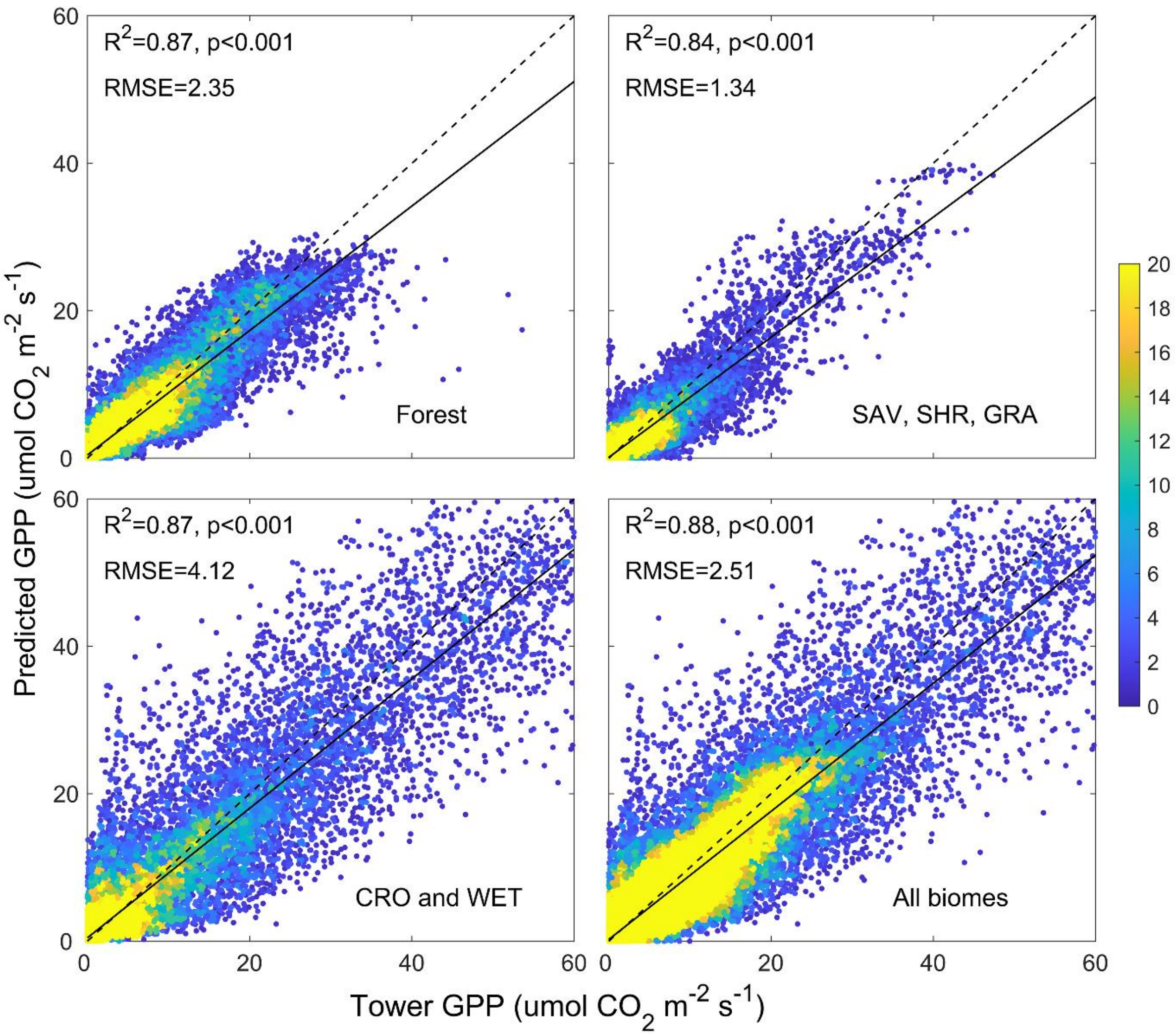

Fig. S24. Evaluation of Cubist model for hourly GPP prediction for different vegetation types.

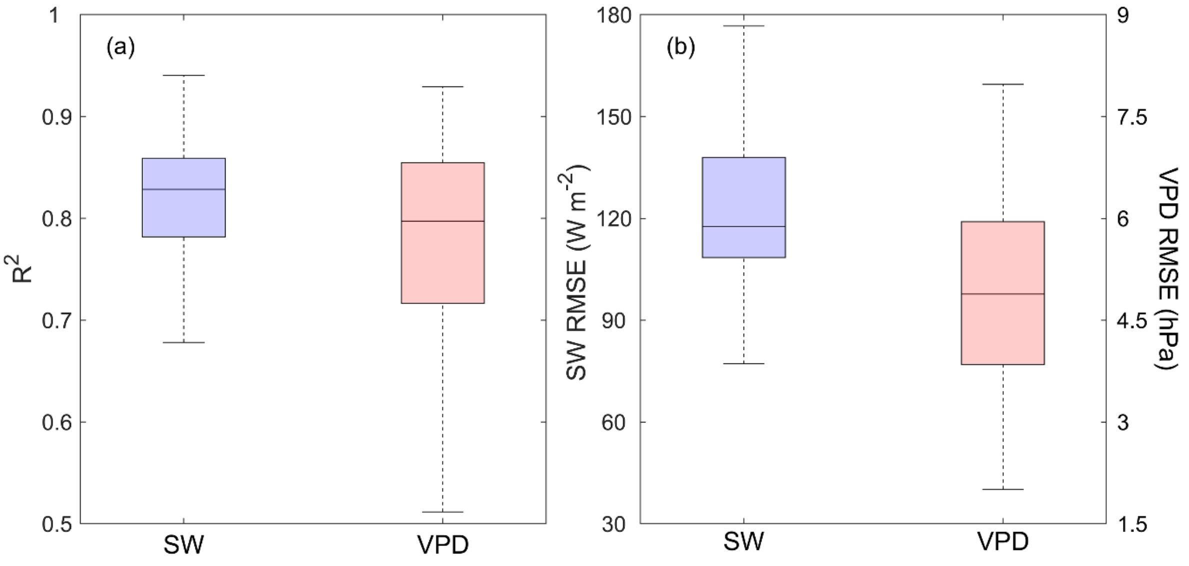

Fig. S25. Evaluation of hourly GOES SW and ERA-5 VPD against tower data. (a, b) show the boxplots of R^2 and RMSE, respectively. The definitions of boxplot elements are the same as those in Fig. S3.

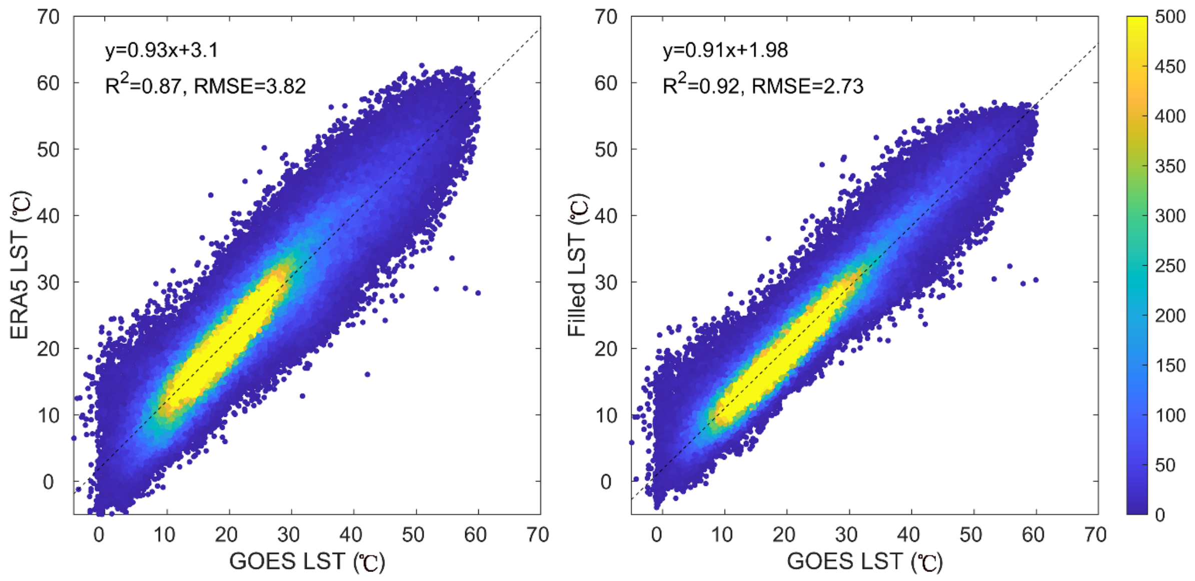

Fig. S26. The scatterplots of GOES-16 LST and ERA5 LST (left) or gap filled GOES-16 LST based on ERA5 LST (right).

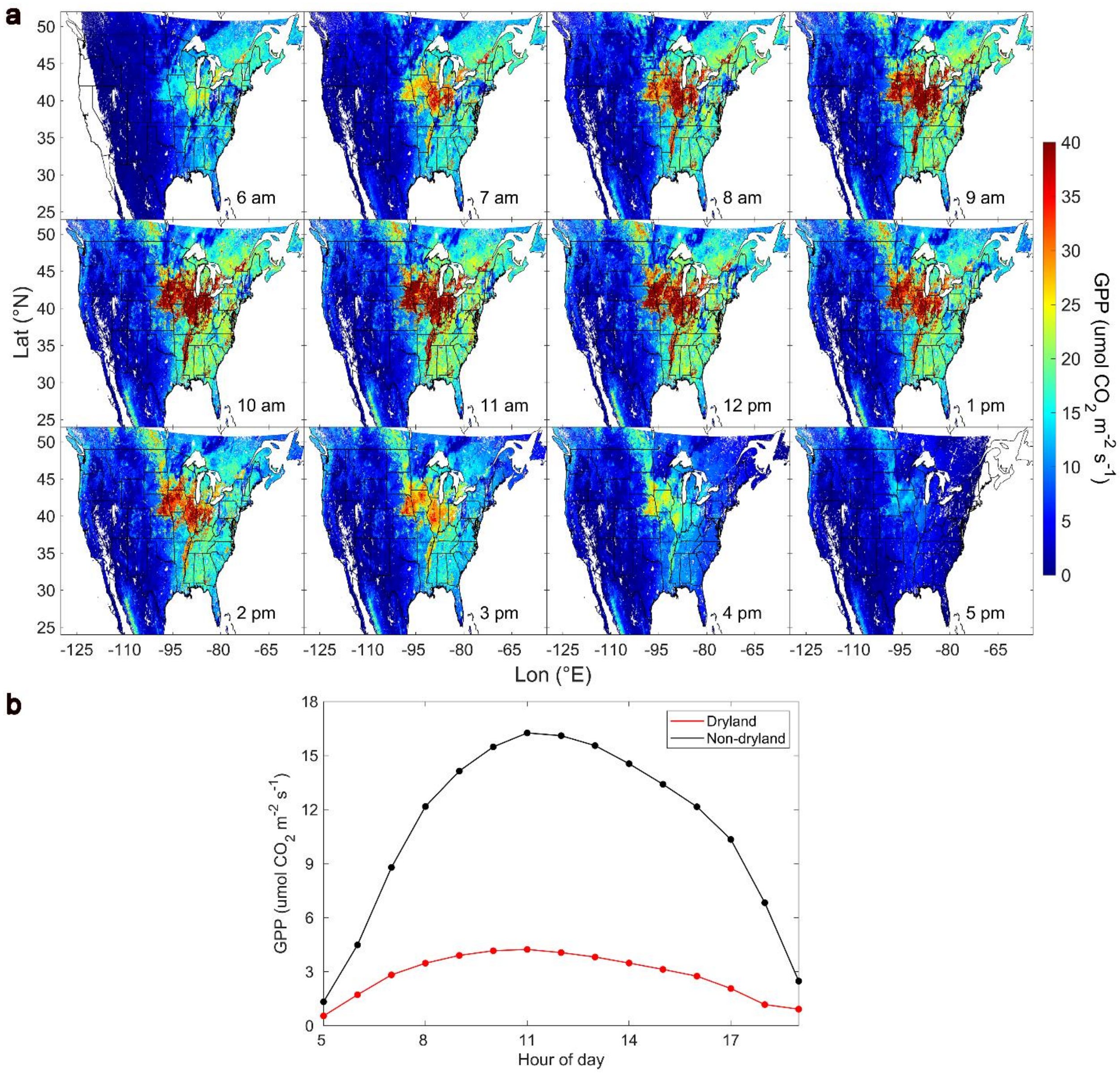

Fig. S27. Example of regional hourly GPP across CONUS from 6 am to 5 pm on August 1, 2018. The hours shown here are indicated by Pacific Daylight Time (PDT) which is 7 hours behind Coordinated Universal Time (UTC). The GPP was predicted by Cubist model based on gridded input variables listed in Table S3. b. shows regional-mean hourly GPP for each hour for dryland and non-dryland. The hours mentioned here correspond to local time.

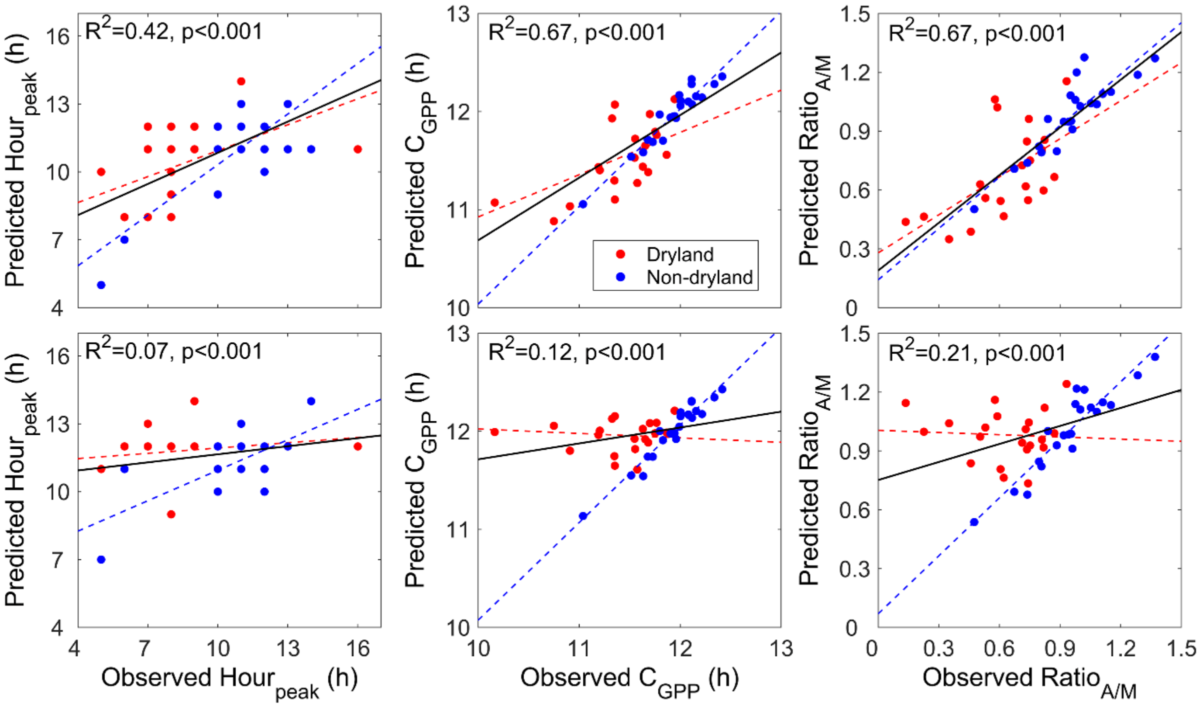

Fig. S28. The evaluation of predicted three diurnal metrics including the diurnal centroid (C_{GPP}), GPP peak hour ($Hour_{Peak}$) and the afternoon GPP/morning GPP ratio ($Ratio_{A/M}$) against that observed by site-level data. The upper panel shows the results for selected model in the study including SW, LST and VPD; while the lower panel shows the results for the tested model only using SW.

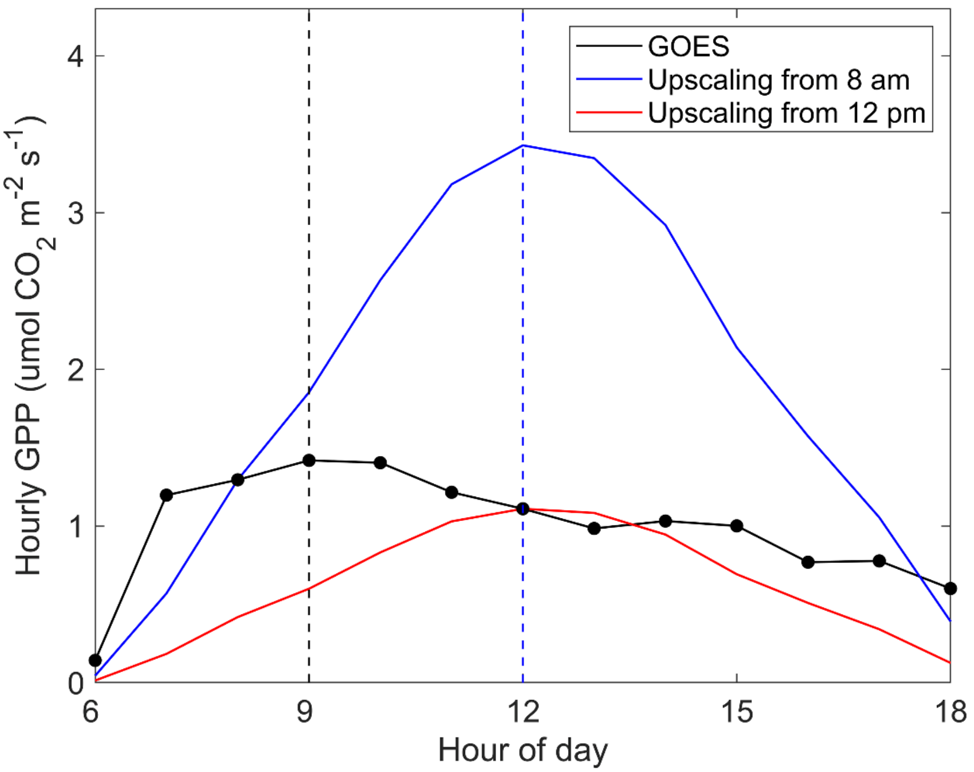

Fig. S29. The illustration of how to upscale instantaneous GPP observations based on the temporal upscaling method proposed by (*19*) at Walnut Gulch Kendall Grasslands (US-Wkg) site. The hourly GPP shown in black is estimated by GOES, while blue and red curves are upscaled based on GPP at 8 am and 12 pm, respectively. The hours mentioned here correspond to local time.

Table S1. List of the data used for training the GPP model

**Explanatory variable**	**Source**	**Temporal resolution**	**Spatial resolution**
Vapor pressure deficit (VPD)	EC flux tower	Half-hourly or hourly	/
Shortwave incoming radiation (SW)	EC flux tower	Half-hourly or hourly	/
Land cover type	EC flux tower	Categorical variable	/
Land surface temperature (LST)	GOES-16	Hourly	~ 2 km
NDVI	MCD43A1	Daily	500 m

Table S2. Details of 77 AmeriFlux sites used in this study. The sites marked with asterisk are used to examine the shift of diurnal metrics during the heatwave shown in Fig. S8. Note that the tower team PI for all the NEON sites (site ID starting with 'US-x') is National Ecological Observatory Network neon-ameriflux@battelleecology.org - NEON Program, Battelle.

**Site ID**	**Site Name**	**Lat**	**Lon**	**Vegetation Type**	**PI**	**DOI**
US-ARM	ARM Southern Great Plains site-Lamont	36.605	-97.4888	CRO	Sebastien Biraud	https://doi.org/10.17190/AMF/1246027
US-Bi2	Bouldin Island corn	38.109	-121.535	CRO	Dennis Baldocchi	https://doi.org/10.17190/AMF/1419513
US-CF1	CAF-LTAR Cook East	46.7815	-117.082	CRO	Dave Huggins; Eric Russell	https://doi.org/10.17190/AMF/1543382
US-CF2	CAF-LTAR Cook West	46.7840	-117.0908	CRO	Dave Huggins; Eric Russell	https://doi.org/10.17190/AMF/1543383
US-CF3	CAF-LTAR Boyd North	46.7551	-117.1261	CRO	Dave Huggins; Eric Russell	https://doi.org/10.17190/AMF/1543385
US-CF4	CAF-LTAR Boyd South	46.7518	-117.1285	CRO	Dave Huggins; Eric Russell	https://doi.org/10.17190/AMF/1543384
US-CS3	Central Sands Irrigated Agricultural Field	44.1394	-89.5727	CRO	Ankur Desai	https://doi.org/10.17190/AMF/1617713
US-DFC	US Dairy Forage Research Center, Prairie du Sac	43.3448	-89.7117	CRO	Alison Duff; Ankur Desai	https://doi.org/10.17190/AMF/1660340
US-GLE	GLEES	41.3665	-106.2399	ENF	Bill Massman<br>John Frank<br>Rob Hubbard	https://doi.org/10.17190/AMF/1246056
US-Ha1	Harvard Forest EMS Tower (HFR1)	42.5378	-72.1715	DBF	J. William Munger	https://doi.org/10.17190/AMF/1246059
US-Ha2	Harvard Forest Hemlock Site	42.5393	-72.1779	ENF	J. William Munger	https://doi.org/10.17190/AMF/1246060
US-HBK	Hubbard Brook Experimental Forest	43.9397	-71.7181	DBF	Eric Kelsey; Mark Green	https://doi.org/10.17190/AMF/1634881
US-Los	Lost Creek	46.0827	-89.9792	WET	Ankur Desai	https://doi.org/10.17190/AMF/1246071

US-Me2	Metolius mature ponderosa pine	44.4523	-121.557	ENF	Bev Law; Chris Still	https://doi.org/10.17190/AMF/1246076
US-Me6	Metolius Young Pine Burn	44.3233	-121.608	ENF	Bev Law; Chris Still	https://doi.org/10.17190/AMF/1246128
US-MMS	Morgan Monroe State Forest	39.3232	-86.4131	DBF	Kim Novick; Rich Phillips	https://doi.org/10.17190/AMF/1246080
US-Mpj*	Mountainair Pinyon-Juniper Woodland	34.4385	-106.238	WSA	Marcy Litvak	https://doi.org/10.17190/AMF/1246123
US-MWA	Jackson Agricultural Field	42.2143	-84.8539	CRO	Jiquan Chen	https://doi.org/10.17190/AMF/1782819
US-MWF	Kellogg Experimental Forest	42.366	-85.3526	MF	Jiquan Chen	https://doi.org/10.17190/AMF/1782820
US-Ne1*	Mead - irrigated continuous maize site	41.1651	-96.47664	CRO	Andy Suyker	https://doi.org/10.17190/AMF/1246084
US-Ne2*	Mead - irrigated maize-soybean rotation site	41.1649	-96.4701	CRO	Andy Suyker	https://doi.org/10.17190/AMF/1246085
US-Ne3*	Mead - rainfed maize-soybean rotation site	41.1797	-96.4397	CRO	Andy Suyker	https://doi.org/10.17190/AMF/1246086
US-NR1*	Niwot Ridge Forest (LTER NWT1)	40.0329	-105.546	ENF	Peter Blanken	https://doi.org/10.17190/AMF/1246088
US-PFa*	Park Falls/WLEF	45.9459	-90.2723	MF	Ankur Desai	https://doi.org/10.17190/AMF/1246090
US-Ro4*	Rosemount Prairie	44.6781	-93.0723	GRA	John Baker; Timothy Griffis	https://doi.org/10.17190/AMF/1419507
US-Ro5*	Rosemount I18_South	44.691	-93.0576	CRO	John Baker; Timothy Griffis	https://doi.org/10.17190/AMF/1419508
US-Ro6*	Rosemount I18_North	44.6946	-93.0578	CRO	John Baker; Timothy Griffis	https://doi.org/10.17190/AMF/1419509
US-Seg*	Sevilleta grassland	34.3623	-106.702	GRA	Marcy Litvak	https://doi.org/10.17190/AMF/1246124
US-Ses*	Sevilleta shrubland	34.3349	-106.744	OSH	Marcy Litvak	https://doi.org/10.17190/AMF/1246125
US-SRG*	Santa Rita Grassland	31.7894	-110.8277	GRA	Russell Scott	https://doi.org/10.17190/AMF/1246154

US-SRM*	Santa Rita Mesquite	31.8214	-110.866	WSA	Russell Scott	https://doi.org/10.17190/AMF/1246104
US-Syv*	Sylvania Wilderness Area	46.242	-89.3477	MF	Ankur Desai	https://doi.org/10.17190/AMF/1246106
US-Ton*	Tonzi Ranch	38.4316	-120.966	WSA	Dennis Baldocchi	https://doi.org/10.17190/AMF/1245971
US-UMB	Univ. of Mich. Biological Station	45.5598	-84.7138	DBF	Christopher Gough; Gil Bohrer; Luke Nave	https://doi.org/10.17190/AMF/1246107
US-UMd	UMBS Disturbance	45.5625	-84.6975	DBF		https://doi.org/10.17190/AMF/1246134
US-Var	Vaira Ranch- Ione	38.4133	-120.951	GRA	Dennis Baldocchi	https://doi.org/10.17190/AMF/1245984
US-Vcm*	Valles Caldera Mixed Conifer	35.8884	-106.532	ENF	Marcy Litvak	https://doi.org/10.17190/AMF/1246121
US-Vcp*	Valles Caldera Ponderosa Pine	35.8642	-106.597	ENF	Marcy Litvak	https://doi.org/10.17190/AMF/1246122
US-Vcs*	Valles Caldera Sulphur Springs Mixed Conifer	35.9193	-106.614	ENF	Marcy Litvak	https://doi.org/10.17190/AMF/1418681
US-WCr*	Willow Creek	45.8059	-90.0799	DBF	Ankur Desai	https://doi.org/10.17190/AMF/1246111
US-Wjs*	Willard Juniper Savannah	34.4255	-105.862	SAV		https://doi.org/10.17190/AMF/1246120
US-Whs*	Walnut Gulch Lucky Hills Shrub	31.7438	-110.052	OSH	Russell Scott	https://doi.org/10.17190/AMF/1246113
US-Wkg*	Walnut Gulch Kendall Grasslands	31.7365	-109.942	GRA	Russell Scott	https://doi.org/10.17190/AMF/1246112
US-xAB	NEON Abby Road (ABBY)	45.7624	-122.33	ENF		https://doi.org/10.17190/AMF/1617726
US-xAE	NEON Klemme Range Research Station (OAES)	35.4106	-99.0588	GRA		https://doi.org/10.17190/AMF/1671891
US-xBR	NEON Bartlett Experimental Forest (BART)	44.0639	-71.2873	DBF		https://doi.org/10.17190/AMF/1579542
US-xCL	NEON LBJ National Grassland (CLBJ)	33.4012	-97.57	GRA		https://doi.org/10.17190/AMF/1671894

US-xCP*	NEON Central Plains Experimental Range (CPER)	40.8155	-104.7456	GRA		https://doi.org/10.17190/AMF/1579720
US-xDC	NEON Dakota Coteau Field School (DCFS)	47.1617	-99.1066	GRA		https://doi.org/10.17190/AMF/1617728
US-xDL	NEON Dead Lake (DELA)	32.5417	-87.8039	MF		https://doi.org/10.17190/AMF/1579721
US-xGR	NEON Great Smoky Mountains National Park, Twin Creeks (GRSM)	35.6890	-83.5020	DBF		https://doi.org/10.17190/AMF/1634885
US-xHA	NEON Harvard Forest (HARV)	42.5369	-72.1727	DBF		https://doi.org/10.17190/AMF/1562391
US-xJE	NEON Jones Ecological Research Center (JERC)	31.1948	-84.4686	ENF		https://doi.org/10.17190/AMF/1617730
US-xJR	NEON Jornada LTER (JORN)	32.5907	-106.8425	OSH		https://doi.org/10.17190/AMF/1617731
US-xKA	NEON Konza Prairie Biological Station - Relocatable (KONA)	39.1104	-96.6130	GRA		https://doi.org/10.17190/AMF/1579722
US-xKZ	NEON Konza Prairie Biological Station (KONZ)	39.1008	-96.5631	GRA		https://doi.org/10.17190/AMF/1562392
US-xMB	NEON Moab (MOAB)	38.2483	-109.3883	PSH		https://doi.org/10.17190/AMF/1671896
US-xML	NEON Mountain Lake Biological Station (MLBS)	37.3783	-80.5248	DBF		https://doi.org/10.17190/AMF/1671897
US-xNG*	NEON Northern Great Plains Research Laboratory (NOGP)	46.7697	-100.915	GRA		https://doi.org/10.17190/AMF/1617732
US-xNQ	NEON Onaqui-Ault (ONAQ)	40.1776	-112.4524	OSH		https://doi.org/10.17190/AMF/1617733
US-xRM	NEON Rocky Mountain National Park, CASTNET (RMNP)	40.2759	-105.546	ENF		https://doi.org/10.17190/AMF/1579723
US-xRN	NEON Oak Ridge National Lab (ORNL)	35.9641	-84.2826	DBF		https://doi.org/10.17190/AMF/1773400
US-xSB	NEON Ordway-Swisher Biological Station (OSBS)	29.6893	-81.9934	ENF		https://doi.org/10.17190/AMF/1671899

US-xSE	NEON Smithsonian Environmental Research Center (SERC)	38.8901	-76.56	DBF		https://doi.org/10.17190/AMF/1617734
US-xSJ	NEON San Joaquin Experimental Range (SJER)	37.1088	-119.7323	SAV		https://doi.org/10.17190/AMF/1671901
US-xSL	NEON North Sterling, CO (STER)	40.4619	-103.0293	CRO		https://doi.org/10.17190/AMF/1617735
US-xSP*	NEON Soaproot Saddle (SOAP)	37.0334	-119.2622	ENF		https://doi.org/10.17190/AMF/1617736
US-xSR*	NEON Santa Rita Experimental Range (SRER)	31.9107	-110.8355	OSH		https://doi.org/10.17190/AMF/1579543
US-xST*	NEON Steigerwaldt Land Services (STEI)	45.5089	-89.5864	DBF		https://doi.org/10.17190/AMF/1617737
US-xTA	NEON Talladega National Forest (TALL)	32.9505	-87.3933	ENF		https://doi.org/10.17190/AMF/1671902
US-xTE	NEON Lower Teakettle (TEAK)	37.0058	-119.006	ENF		https://doi.org/10.17190/AMF/1617738
US-xTR*	NEON Treehaven (TREE)	45.4937	-89.58571	DBF		https://doi.org/10.17190/AMF/1634886
US-xUK	NEON The University of Kansas Field Station (UKFS)	39.0404	-95.1921	DBF		https://doi.org/10.17190/AMF/1617740
US-xUN*	NEON University of Notre Dame Environmental Research Center (UNDE)	46.2339	-89.5373	MF		https://doi.org/10.17190/AMF/1617741
US-xWD	NEON Woodworth (WOOD)	47.1282	-99.2414	GRA		https://doi.org/10.17190/AMF/1579724
US-xWR	NEON Wind River Experimental Forest (WREF)	45.8205	-121.9519	ENF		https://doi.org/10.17190/AMF/1617742
US-xYE	NEON Yellowstone Northern Range (Frog Rock) (YELL)	44.9535	-110.539	ENF		https://doi.org/10.17190/AMF/1617743

Table S3. List of the data used for regional hourly GPP prediction

Explanatory variable	Source	Temporal resolution	Spatial resolution
Shortwave incoming radiation (SW)	GOES-16	Hourly	0.25°
Land surface temperature (LST)	GOES-16	Hourly	~ 2 km
VPD and LST	ERA5	hourly	0.1°
NDVI	MCD43C4	Daily	0.05°
Land cover type	MCD12C1	Categorical variable	0.05°

Table S4. Statistical measures for model evaluation with GPP derived from daytime partitioning method. The selected model is marked with asterisk.

Training			Testing		Variable combinations
**AE**	**RE**	**r**	**R^2**	**RMSE**	
1.09	0.26	0.93	0.88	2.51	**VPD SW NDVI LST Biome** *
1.09	0.26	0.93	0.88	2.47	VPD SW EVI LST Biome
1.10	0.26	0.92	0.88	2.49	VPD SW NIRv LST Biome
2.11	0.50	0.82	0.65	4.29	VPD NDVI LST Biome, without SW
2.03	0.48	0.74	0.54	4.86	VPD SW LST Biome, without NDVI
1.50	0.36	0.85	0.77	3.42	VPD SW NDVI LST, without Biome
1.12	0.27	0.93	0.87	2.60	VPD SW NDVI Biome, without LST
1.09	0.26	0.93	0.87	2.57	SW NDVI LST Biome, without VPD
1.20	0.29	0.93	0.86	2.63	SW NDVI Biome, without VPD and LST

Table S5. Statistical measures for model evaluation with GPP derived from (a) nighttime partitioning method and (b) GPP based on different sampling methods (different percentage of high LST samples). In the second test, we increased the proportion of samples with higher LST. Model 1: selected model with evenly distributed LST; Model 2: only used 1/2 samples of Model 1 with LST < 20°, 3/4 samples with LST ~ [20°, 30°), and 100% samples with LST >= 30°; Model 3: only used 1/3 samples of Model 1 with LST < 20°, 1/2 samples with LST ~ [20°, 30°), and 100% samples with LST >= 30°.

**(a)**	**Training**			**Testing**		**Variable combinations**
	**AE**	**RE**	**r**	R^2	**RMSE**	
	1.47	0.33	0.92	0.86	2.82	VPD SW NDVI LST Biome
	2.33	0.52	0.81	0.66	4.40	VPD NDVI LST Biome, without SW
	2.38	0.51	0.75	0.55	5.01	VPD SW LST Biome, without NDVI
	1.87	0.42	0.84	0.77	3.57	VPD SW NDVI LST, without Biome
	1.49	0.34	0.92	0.85	2.90	VPD SW NDVI Biome, without LST
	1.46	0.33	0.92	0.85	2.87	SW NDVI LST Biome, without VPD
**(b)**	1.09	0.26	0.93	0.88	2.51	Model 1
	1.27	0.27	0.93	0.88	2.51	Model 2
	1.39	0.29	0.92	0.87	2.52	Model 3

Table S6. Data variables and access links

Data variables	Link
GOES-16 SW and LST	https://www.class.noaa.gov
AmeriFlux	https://ameriflux.lbl.gov
MODIS land cover map	https://e4ftl01.cr.usgs.gov/MOTA/MCD12C1.006/
MODIS BRDF-corrected reflectance	https://e4ftl01.cr.usgs.gov/MOTA/MCD43C4.006/
ERA5-land hourly VPD and LST	https://cds.climate.copernicus.eu/
MERRA-2 daily air temperature and VPD	https://disc.gsfc.nasa.gov
Global aridity map	https://cgiarcsi.community/data/global-aridity-and-pet-database/
